# Identification and Characterization of a New Thermophilic κ-Carrageenan Sulfatase

**DOI:** 10.1021/acs.jafc.4c09751

**Published:** 2025-01-11

**Authors:** Nanna Rhein-Knudsen, Diego S. Reyes-Weiss, Leesa J. Klau, Alexandra Jeudy, Thomas Roret, Runar Stokke, Vincent G. H. Eijsink, Finn L. Aachmann, Mirjam Czjzek, Svein Jarle Horn

**Affiliations:** † Faculty of Chemistry, Biotechnology, and Food Science, NMBU Norwegian University of Life Sciences, P.O. Box 5003, 1432 Aas, Norway; ‡ CNRS, Integrative Biology of Marine Models, Station Biologique de Roscoff, Sorbonne Universitée, 29680 Roscoff, France; § Department of Biotechnology and Food Science, NTNU Norwegian University of Science and Technology, Sem Sælands vei 6/8, 7419 Trondheim, Norway; ∥ Department of Process Technology, SINTEF Industry, Forskningsveien 1, 0373 Oslo, Norway; ⊥ Department of Biological Sciences and Centre for Deep Sea Research, University of Bergen, 5020 Bergen, Norway

**Keywords:** sulfated carbohydrate, carrageenan modification, carbohydrate sulfatase, substrate specificity, 4-O-sulfatase

## Abstract

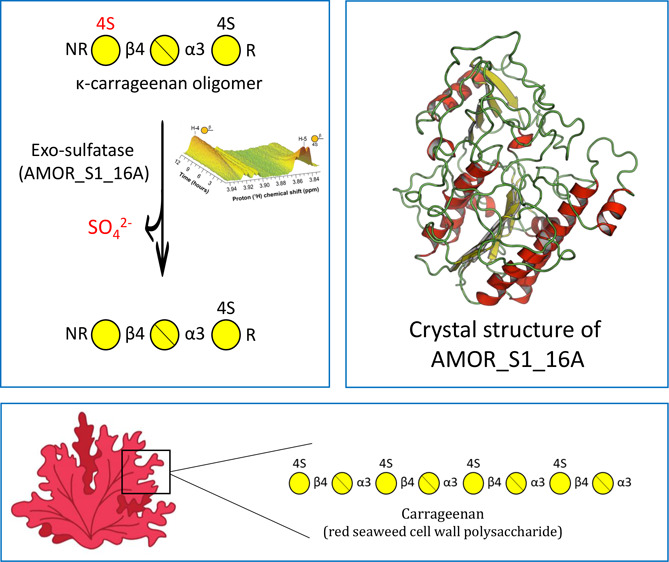

Carrageenans are sulfated polysaccharides found in the cell wall of certain red seaweeds. They are widely used in the food industry for their gelling and stabilizing properties. In nature, carrageenans undergo enzymatic modification and degradation by marine organisms. Characterizing these enzymes is crucial for understanding carrageenan utilization and may eventually enable the development of targeted processes to modify carrageenans for industrial applications. In our study, we characterized a κ-carrageenan sulfatase, AMOR_S1_16A, belonging to the sulfatase S1_16 subfamily, which selectively desulfates the nonreducing end galactoses of κ-carrageenan oligomers in an exomode. Notably, AMOR_S1_16A represents the first κ-carrageenan sulfatase within the S1_16 subfamily and exhibits a novel enzymatic activity. This study provides further understanding of the substrate specificity and characteristics of the S1_16 subfamily. Moreover, this research highlights that many processes and enzymes remain to be discovered to fully understand carrageenan utilization pathways and to develop enzymatic processes for carrageenan modification and processing.

## Introduction

Carrageenans are sulfated polysaccharides found in the cell wall of certain red algae and consist of a backbone of galactose (G) residues linked together by alternating α-1,3 and β-1,4 glycosidic bonds. Various types of carrageenans exist, distinguished by the position and degree of sulfate substitutions, as well as the presence of 3,6-anhydrogalactose (DA), which is unique to red seaweeds.^1^ The commercially important carrageenans include κ-, ι-, and λ-carrageenan. κ-carrageenan contains DA and galactose-4-sulfate (G4S), ι-carrageenan includes 2-O sulfated DA and G4S, while λ-carrageenan comprises galactose-2-sulfate and 2,6-disulfated galactose as the main repeating units.^1,2^ In nature, carrageenans are enzymatically degraded by marine organisms that have evolved specific glycosyl hydrolases (GH) and sulfatases for this purpose. These enzymes were recently reviewed by Jiang et al.^3^ Enzymes involved in the breakdown of the carrageenan backbone include endoacting carrageenases that cleave the internal β-1,4 linkages to produce carrageenan oligomers and are classified in the CAZy database^4^ as the GH families GH16 (κ-carrageenases), GH82 (mainly ι-carrageenases), and GH150 (λ-carrageenases). Exoacting carrageenan degrading enzymes attacking the β-linkages belong to the GH families GH2 (β-galactosidases) and GH167 (exoacting β-carrageenases). Enzymes responsible for the hydrolyzing the α-1,3 linkages have been identified from the GH127 (α-1,3-anhydro-galactosidases) and the GH129 (α-1,3-anhydro-galactosidases) families.^4,5^ Sulfatases, which remove sulfate esters, modify the functional properties of carrageenan as well as its susceptibility to various GH enzyme types. They are classified into 4 families based on sequence homology, structure and mechanism (SulfAtlas), where the S1 formyl-glycine dependent sulfatase family include most of known sulfatases.^6,7^ Sulfatases acting on marine polysaccharides have been extensively reviewed recently by Hettle et al.^8^ To date, only a few carrageenan-active sulfatases have been characterized and they belong to the S1 subfamilies 7, 17, 19, and 81.^6−8^ The first reported carrageenan sulfatase is the exoacting κ-carrageenan sulfatase Psc-κ-Cgs from *Pseudomonas carrageenovora*, which removes the 4-O-sulfate ester at the nonreducing end of κ-carrageenan oligomers.^9,10^ Subsequently, an endoacting 4S-ιota-carrageenan sulfatase from *Paraglaciecola atlantica* (previously classified as *Pseudoalteromonas atlantica*), PaS1_19A, specific for removal of internal 4-O-sulfate esters in ι-carrageenan, was identified.^11^ Two endoacting κ-carrageenan sulfatases, Q15XH1_S1_7 and Q15XG7_S1_19, have also been identified from *P. atlantica*.^7,12^ Highlighting the specificity of carrageenan sulfatases, it has been shown that the 4S-ιota-carrageenan sulfatase PaS1_19A does not remove sulfate esters at position 4 in κ-carrageenan,^11^ while the Q15XH1_S1_7 and Q15XG7_S1_19 κ-carrageenan sulfatases do not show activity on ι-carrageenan.^12^ In contrast to the first identified carrageenan sulfatase (Psc-κ-Cgs) that acted on oligomers only, the three sulfatases identified from *P. atlantica* are also acting on the carrageenan polymers, highlighting the diversity of sulfatases in the S1 family.^11,12^ The enzymatic apparatus involved in carrageenan degradation has been characterized in several marine microbes, for example *Zolbellia galactanivorans* and *Pseudoalteromonas fuliginea* PS47, demonstrating the necessity for several sulfatases with varying substrate preferences.^5,13^

S1 sulfatases are calcium-dependent and contain an essential calcium binding site (typically D-D-D-Q/N) conserved across all S1 sulfatases with known 3D structure. They require post-translational modification of a cysteine or serine to formyl-glycine, which is catalyzed by the formyl-glycine generating enzyme. This modification is directed by a 12 amino acid sequence (C/S-X-P/A-X-R-X-X-X-L/X-T/X-G/X-R/X) highly conserved within the family, beginning with the C/S that undergoes modification. Additionally, S1 sulfatases share a set of polar residues in the catalytic site crucial for sulfate hydrolysis.^8,14^ Despite advances in sequence analysis, accurately predicting substrate specificity remains challenging due to limited data on critical residues involved in substrate recognition.

Given the prevalence of sulfated polysaccharides like agar, carrageenans, fucoidans, and ulvans in seaweeds, many marine microbes possess the metabolic machinery needed for the utilization of these biopolymers, thus playing crucial roles in the marine carbon cycle. While numerous enzymes active on such polysaccharides originating from marine microorganisms and environments have been characterized,^11,15−18^ information regarding marine polysaccharide-specific sulfatases remains limited. Nevertheless, the significance of these enzymes is evident, particularly in industrial processing of carrageenans, where targeted removal of sulfate groups and the production of new carrageenan structures can generate new functional properties and potential bioactivities.

Here, we describe the identification, production and functional – and structural characterization of a thermostable carrageenan sulfatase identified from a metagenomic data set collected from the Arctic Mid-Ocean Ridges (AMOR). Thermophilic enzymes hydrolyzing the brown seaweed polysaccharide alginate have previously been discovered in this data set.^18,19^ The production of sulfated exopolysaccharides has also been described for deep-sea hydrothermal microbes,^20,21^ hence making these habitats promising for discovery of novel sulfatases.

Named AMOR_S1_16A, this enzyme exhibits an exoacting mechanism, removing the sulfate ester at position 4 of the nonreducing end of κ-carrageenan oligomers. AMOR_S1_16A represents the first identified and structurally solved κ-carrageenan sulfatase from the S1_16 subfamily. With only a limited number of available structures, it introduces an important novel functionality to the diverse and complex enzyme repertoire for carrageenan processing, while enhancing our understanding of the substrate specificity and characteristics of the S1_16 subfamily.

## Materials and Methods

### Sampling, DNA Extraction, and Sequencing

A targeted deep-sea hydrothermal in situ enrichment was conducted using a sample of unbleached Norway spruce (*Picea abies*) that had been pretreated by sulfite-pulping using the BALI process developed at Borregaard AS (Sarpsborg, Norway).^22,23^ Further details of the substrate and incubation have been described elsewhere.^24,25^ In brief, 1 g of spruce material was mixed with approximately 16 mL of sediment sampled at the vent site and placed in the middle chamber of a titanium incubator (CGB6.2) with three vertically aligned chambers of 2.5 cm in length, a volume of 16 mL and 1 mm pores. The incubator was deployed for one year in ∼70 °C hot sediments in the Bruse vent field at the AMOR, 570 m below sea level.

DNA was extracted from 4.6 g of material using the FastDNA spin kit for soil (MP Biomedicals, Santa Ana, CA), according to the manufacturer’s protocol. Metagenomic sequencing of total DNA was carried out using Illumina MiSeq 300 paired-end chemistry at the Norwegian Sequencing Centre (www.sequencing.uio.no). 1.8 μg of DNA was submitted for sequencing.

### In Silico Metagenomic Screening

Details on metagenomic filtering, assembly, and open reading frame (ORF) prediction have been described elsewhere.^24^ Predicted ORFs were initially mined for putative GHs using the standalone dbCAN annotation tool (run_dbCAN 3.0) for automated CAZyme annotation.^26^ The script was adjusted to include the SulfAtlas (v1.1) database^6,7^ for detection of sulfatases. As implemented in run_dbCAN.py, diamond blastp (v2.0.13)^27^ was run against the CAZydb (v07312019)^28^ and the SulfAtlas (v1.1)^6,7^ using an e-value cutoff of 1 × 10^–102^. The hmmscan (HMMER v3.1b2)^29^ was executed using dbCAN release 9^30^ with an e-value cutoff of 1 × 10^–15^ and coverage = 0.35. Resulting hits from run_dbCAN.py were subjected to a diamond blastp (v2.0.13)^27^ homology search using Uniref90 (release august_2020)^31^ and signal peptide predictions using a standalone version of the Signalp5.0^32^ with combined search against both archaea, Gram-positive and Gram-negative bacteria. Multiple sequence alignment (MSA) of AMOR_S1_16A and other sulfated polysaccharide sulfatases was performed using the Clustal Omega service^33^ and Jalview software.^34^ InterProScan^35^ was used for functional domain searches.

### Protein Production

The gene encoding AMOR_S1_16A, without the predicted N-terminal signal peptide, was codon optimized for expression in *Escherichia coli*, synthesized by GenScript (Piscataway, NJ, USA) and cloned into the pET28b(+) vector between the *Nco*I and *Xho*I restriction sites with a C-terminal His_6_-tag. Expression was performed in *E. coli* BL21(DE3) cells (Invitrogen) grown in Terrific Broth medium overnight at 18 °C and induced at OD ≈ 0.8 using 1 mM IPTG (Protein Ark, Sheffield, UK) for protein induction. Cells were harvested by centrifugation at 4000 *g*, 20 min, 4 °C (Megastar 1.6R, VWR, Germany) and frozen to promote cell lysis. The following day, the cells were resuspended in 50 mM Tris-HCl, pH 7.4, 500 mM NaCl, 20 mM Imidazole and sonicated on ice (Sonics & Materials Inc., Newtown, Connecticut, USA). Cell debris was removed by centrifugation at 12,500 *g* for 20 min (Megastar 1.6R, VWR, Germany) and the cell-free extract was filtered using a 0.22 μm cutoff before purification of the protein by immobilized metal affinity chromatography, using a BioRad chromatography system equipped with a Ni^2+^ affinity HisTrap FF 5 mL column (GE HealthCare, Chicago, USA). Elution was done with a linear gradient of 20–500 mM Imidazole in 50 mM Tris-HCl, pH 7.4, 500 mM NaCl. Protein-containing fractions were analyzed by SDS-PAGE (BioRad, Hercules, CA, USA) and fractions containing the enzymes were pooled together before imidazole was removed and the buffer was exchanged to 25 mM NaOAc, pH 5.6, 200 mM NaCl using a HiPrep 26/10 desalting column (Cytiva, Sweden). Protein concentrations were determined by A280 absorbance measurements (Synergy H4 HybridReader, BioTek) and using the theoretical extinction coefficient of AMOR_S1_16A (https://web.expasy.org/protparam/).

Sulfatase activity was verified using the model substrate 4-nitrocatechol sulfate dipotassium salt (pNCS) that changes color when sulfate is cleaved off. 10 μM AMOR_S1_16A was incubated overnight with 10 mM pNCS (Sigma-Aldrich) in 25 mM NaOAc, pH 5.6, 200 mM NaCl at 40 and 60 °C. Reactions were stopped with 1 M NaOH and color development, as a result of sulfate removal, was measured at 515 nm.

### Biochemical Characterization

The effect of temperature on enzymatic activity was assayed by incubating 1 μM AMOR_S1_16A with 2.5 mM pNCS in 25 mM NaOAc, pH 5.6, 300 mM NaCl, at temperatures varying from 25 to 90 °C for 40 min. The pH optimum was determined covering a pH range from 3.6 to 9 using 1 μM AMOR_S1_16A with 2.5 mM pNCS in either 25 mM NaOAc or 25 mM Tris-HCl (Note: pH was measured at 60 °C) for 40 min. The effect of NaCl was measured from 0 to 2000 mM with 1 μM AMOR_S1_16A and 2.5 mM pNCS in 25 mM NaOAc, pH 5.6 at 60 °C in reactions of 40 min. The influence of cations on sulfatase activity was determined after preincubation of 10 μM AMOR_S1_16A with 10 mM MnCl_2_, NiCl_2_, ZnCl_2_, CaCl_2_, CuCl_2_, or MgCl_2_ for 10 min followed by reaction with 2.5 mM pNCS in 25 mM NaOAc, pH 5.6, 400 mM NaCl, at 60 °C for 40 min. The effect of CaCl_2_ was further tested at 2.5 mM and 5 mM to determine the optimal calcium concentration for activity (2.5 mM pNCS in 25 mM NaOAc, pH 5.6, 400 mM NaCl, at 60 °C). Before testing the effects of cations, AMOR_S1_16A was first incubated with 1 mM EDTA for 15 min to exclude the possibility of any bound metals prior to the analysis. EDTA was subsequently removed by dialysis. Thermostability was evaluated by measuring residual sulfatase activity (1 μM AMOR_S1_16A with 2.5 mM pNCS in 25 mM NaOAc, pH 5.6, 400 mM NaCl, 10 mM CaCl_2_ at 60 °C) after preincubation of the enzyme in reaction buffer without substrate at 60 °C for 0–24 h. Product formation was measured after 40 min reaction. The thermostability of AMOR_S1_16A was additionally evaluated by preincubating the enzyme at different temperatures for 16 h.

### Substrates

Polymeric, oligomeric, and monomeric sulfated carbohydrates were used as substrates to determine the specificity of AMOR_S1_16A. κ-carrageenan (Tokyo Chemical Industry), ι-carrageenan (Tokyo Chemical Industry), λ-carrageenan (Tokyo Chemical Industry), and agar (Sigma-Aldrich) were tested in their native form and as hydrolysates. To generate a variety of substrates, partial hydrolysis of these polysaccharides was performed with 1 M TFA at 60 °C for 1 h, followed by neutralization with 5% NH_4_OH. Additionally, κ-carrageenan oligomers were enzymatically produced using the κ-carrageenase ZgCgk16A from *Z. galactanivorans*(36) (EC number 3.2.1.83) (NZYTech, Portugal) using 10 mg/mL substrate and 1 μL enzyme (0.25 mg/mL) in 50 mM Tris-HCl, pH 7.2, 150 mM NaCl at 25 °C overnight. In addition, AMOR_S1_16A was tested on a wide range of commercial fucoidan substrates: *Macrocystis pyrifera*, *Undaria pinnatifida*, *Fucus vesiculosus*, *Laminaria japonica* from Sigma-Aldrich, and *Alaria* sp. *Fucus serratus*, *Laminaria digitata*, *Ascophyllum nodosum*, *Lessonia nigrescens*, *Ecklonia* sp., *Durvillaea* sp., *Cladosiphon* sp. from Biosynth. Fucoidan substrates were used in their native form and as hydrolysates, as described above for agar and carrageenans. The monosaccharides galactose-4-sulfate (G4S) and galactose-6-sulfate (G6S) were obtained from Biosynth Ltd., UK, and *N*-acetylgalactosamine-4-sulfate (4S-GalNAc) from Dextra, UK (Kindly provided by Dr. Alan Cartmell, Newcastle University, UK), respectively.

### Size Exclusion Chromatography

The production of sulfated oligosaccharides was confirmed using size-exclusion chromatography (SEC-RI) on an Ultimate3000 system (Dionex, Sunnyvale, USA) coupled with a RI-detector (RefractoMax520, ERC) as described previously.^37^ In brief, 50 μL of a 5–10 mg/mL solutions of native polysaccharide and oligomers were injected into a setup consisting of a TSKgelPWXL guard column (6 mm × 4 cm, 12 μm particle size) connected in series to a TSKgelG4000PWXL column (7.8 mm × 30 cm, 10 μm particle size) and a TSKgelG5000PWXL column (7.8 mm × 30 cm, 10 μm particle size). Elution was performed with 0.15 M NaNO_3_, 0.01 M EDTA, pH 6.0, at a flow rate of 0.5 mL/min and pullulan standards with molecular weights ranging from 1.3 to 800 kDa were used.

### MALDI-TOF Mass Spectrometry

Further confirmation of sulfated oligomers was carried out using Matrix Assisted Laser Desorption Ionization-Time of Flight (MALDI-TOF) mass spectrometry with an Ultraflextreme MALDI-TOF mass spectrometer (Bruker) in the reflectron mode, employing conditions previously reported for the analysis of enzymatic hydrolysates of carrageenan.^38^ Samples were diluted to 0.1 mg/mL in 100 mM NaCl, mixed 1:1 with matrix solution (2.5 mg/mL norharmene in EtOH/H_2_O (1:1 v/v), 0.1% (v/v) TFA), and 1 μL of the mixture was deposited and air-dried on a steel plate (MTP 384 target plate ground steel BC, Bruker Daltonics).

### Enzyme Specificity Assays

To determine the substrate specificity of AMOR_S1_16A, 1 μM enzyme was incubated with 1 mg/mL substrate under optimal reaction conditions (25 mM NaOAc, pH 5.6, 400 mM NaCl, 10 mM CaCl_2_ at 60 °C) for 24 h. Sulfate release was detected by high performance anion exchange chromatography (HPAEC) using an ICS-6000 chromatography system (Dionex) equipped with an ED40 electrochemical detector. Ions were separated on an AS11-HC anion-exchange column (2 × 250 mm; Dionex) with accompanying AG11-HC guard column (2 × 50 mm; Dionex) and elution was done with 5 mM KOH using an isocratic flow rate of 0.4 mL/min. Background signal and noise originating from the eluent was reduced using an anion self-regenerating suppressor (AERS-500, Dionex) with a current of 5 mA. Sulfate concentrations were quantified using a K_2_SO_4_ calibration curve.

### Nuclear Magnetic Resonance Spectroscopy

All homo- and heteronuclear experiments were recorded on a Bruker 800 MHz Avance III HD spectrometer (Bruker BioSpin AG, Fällanden, Switzerland) equipped with a 5 mm cryogenic CP-TCI z-gradient probe and processed using TopSpin versions 3.5 and 4.3.0 (Bruker BioSpin AG). Proton chemical shifts were internally referenced to the residual water signal (4.75 ppm at 25 °C and 4.50 ppm at 50 °C) and carbon chemical shifts were indirectly referenced to DSS (2,2-dimethyl-2-silapentane-5-sulfonic acid) using a ^13^C/^1^H frequency ratio of 0.251449530.^39^

To determine the structural changes in κ-carrageenan oligomers after treatment with AMOR_S1_16A, enzyme reaction and control samples were analyzed and compared with 2D nuclear magnetic resonance (NMR) spectroscopy. Reactions were performed in 1 mL total volume overnight with 10 μM AMOR_S1_16A and 2 mg/mL TFA-hydrolyzed κ-carrageenan substrate under optimal reaction conditions (25 mM NaOAc, pH 5.6, 400 mM NaCl, 10 mM CaCl_2_ at 60 °C). Prior to analysis, the reaction mixtures were desalted and buffer exchanged to 100 mM MES, pH 5.6, 250 mM NaCl, using a PD-10 column (Cytvia Life Sciences), before being freeze-dried. Freeze-dried reaction products were resuspended in 200 μL of 99.9% D_2_O to reduce the water signal in NMR (giving approximately 10 mg/mL substrate concentration) and transferred to 3 mm 4” NMR tubes (LabScape). The following experiments were recorded for each sample: 1D proton spectrum with water suppression (noesygppr1d), ^1^H–^13^C heteronuclear single quantum coherence (HSQC) spectrum with multiplicity editing (hsqcedetgpsisp2.3), ^1^H–^13^C heteronuclear two bond correlation (H2BC) spectrum (h2bcetgpl3pr), ^1^H–^13^C heteronuclear multiple bond coherence (HMBC) spectrum with suppression of one-bond correlations (hmbcetgpl3nd), ^1^H–^1^H in-phase correlation spectroscopy (IP-COSY) (ipcosyesgp-tr), H–^1^H total correlation spectroscopy (TOCSY) with 70 ms mixing time (clmlevphpr), and ^1^H–^1^H nuclear Overhauser effect spectroscopy (NOESY) with 80 ms mixing time (noesyesgpph) and the homonuclear spectra use excitation sculpting water suppression.

To monitor the reaction over time, a mixture was prepared by combining 180 μL of 10 mg/mL TFA-hydrolyzed κ-carrageenan in 10 mM NaOAc, 200 mM NaCl, 10 mM CaCl_2_, pH 5.6 in 99.9% D_2_O with 20 μL of a 320 μM solution of AMOR_S1_16A in H_2_O, giving a final concentration of 32 μM. After adding the enzyme, a pseudo-2D experiment was recorded at 50 °C consisting of a series of 1D ^1^H spectra with water suppression (noesygppr1d, ns = 24) collected every 5 min for a total of 144 spectra (total experiment time 12 h). Subsequently, a ^1^H–^13^C HSQC spectrum with multiplicity editing was recorded.

### Crystallization and Structure Determination

The AMOR_S1_16A sulfatase was expressed and purified as described above. After His-tag purification, AMOR_S1_16A was further purified using a HiLoad Superdex 200 column (Cytvia Life Sciences) with 25 mM NaOAc, pH 5.6, 100 mM NaCl, 5 mM CaCl_2_, as the running buffer. AMOR_S1_16A was concentrated to 7.9 mg/mL using a 10 kDa filter (Vivaspin, Sartorius). Crystallization screening was performed with a Crystal Gryphon robot (Art Robbins Instruments, Hudson) with the following commercial protein crystallization kits: PACT Suite (Qiagen), Classic Suite (Qiagen), and JCSG+ (Molecular Dimensions). Crystallization trials were set up in sitting drops, by mixing 0.2 μL of enzyme solution with 0.1 μL of reservoir solution, and equilibration against 80 μL of reservoir solution. Initial crystallization conditions that were identified in the screening conditions were further optimized in 24-well plates using the hanging drop vapor-diffusion method, by mixing 2 μL protein solution with 1 μL reservoir solution and equilibrating against 500 μL reservoir solution. The best crystals were obtained in 0.1 M Sodium citrate at pH 5.0 containing 12% Polyethylene-glycol (PEG) 3350. Crystals were transferred into a drop of mother liquor containing 15% glycerol as cryo-protectant, subsequently flash-frozen in liquid nitrogen and stored in an Unipuck device for transport to the SOLEIL synchrotron (St Aubin, France). Diffraction data were collected at 100 K on beamline Proxima 2 and processed using XDS^40^ and Aimless from the CCP4 program package.^41^ The structure of AMOR_S1_16A was solved by molecular replacement with the Phenix suite program Phaser^42^ using the model produced by Alphafold2^43^ as the starting model. Iterative rounds of model building and refinement were carried out using Coot^41^ and the phenix.refine module of PHENIX.^44^ Validation of the crystal structure was performed with MolProbity.^45^ Data collection and refinement statistics are provided in Supplementary Table S1.

### Molecular Docking and Structure Impositions

All crystal structures were superimposed using the secondary-structure matching (SSM) routine of Coot^41^ and structural figures were produced with PyMOL (The PyMOL Molecular Graphics System, Version 1.2r3pre, Schrödinger, LLC.). The glycerol and 4S-GalNAc molecules, located in the active site of AMOR_S1_16A and BT3057-S1_16 (pdb entry 7OZ9), respectively, were used as a guide to dock the neo-κ-carratetraose into the active site of AMOR_S1_16A.

### Data Availability

The sequence of AMOR_S1_16A has been submitted to GenBank under accession number PP524981 and archived under BioProject PRJNA296938 and BioSample SAMN09768205. The crystal structure presented has been deposited with PDB entry 9FO1.

## Results and Discussion

### Identification of a Sulfatase

To identify new seaweed carbohydrate sulfatases, the AMOR metagenome was screened for genes encoding carbohydrate active enzymes. A total of 16,106 ORFs had significant hits to dbCAN, CAZy, or SulfAltas (or multiple hits to each), with 473 of those having hits to SulfAtlas.

**Figure 1:**
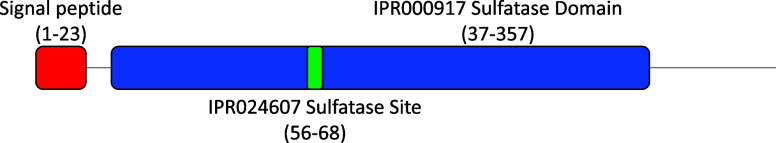
Primary structure and predicted domains of AMOR_S1_16A. Numbering corresponds to the full-length enzyme sequence and identified domains are indicated as follows: red: N-terminal signal peptide; blue: the IPR000917 N-terminal sulfatase domain; and green: the IPR024607 sulfatase site.

Originally, this study was started with the objective to identify new fucoidan-acting sulfatases, and AMOR_S1_16A was selected based on both the sulfatase subfamily and the gene location. Based on a recent study on the seaweed degrading marine bacterium Verrucomicrobia *Lentimonas* sp.CC4, that showed that a surprisingly high number of S1_16 sulfatases were present in its genome,^46^ the S1_16 subfamily was found interesting as no enzymes from this family have been identified with activity on seaweed polysaccharides. 52 out of the 473 hits to SulfAtlas were sulfatases appointed to the S1_16 subfamily. AMOR_S1_16A, linked to a metagenome-assembled genome from the family *Bryobacteraceae,* is located on the same contig as genes encoding GHs from families that, among other, contain putative enzymes acting on seaweed polysaccharides. These families include GH family 29, 95, 109, 116, 141, and 151, from which fucoidan-active enzymes have been characterized from the GH families 29 and 95.^47^ The AMOR_S1_16A protein is 485 amino acids long and contains a predicted N-terminal signal peptide of 23 amino acids. InterProScan analysis revealed several predicted conserved domains and residues in the protein sequence. The N-terminal sulfatase domain IPR000917 was predicted from position 37 to 357 including the conserved sulfatase site IPR024607 that starts with the cysteine normally modified to form formyl-glycine, Figure 1.

Sulfatase subfamily assignment was based on protein BLAST using SulfAtlas.^7^ AMOR_S1_16A belongs to the sulfatase S1_16 subfamily, which includes known activities for *N*-acetylgalactosamine-6-sulfate (6S-GalNAc) and G4*S*/4S-GalNAc.^14,48^ AMOR_S1_16A has the highest sequence identity (68%) to a S1_16 subfamily DUF4976 domain-containing protein identified in a metagenomic data set collected from sulfur-rich hydrothermal sediments in the South Atlantic Ocean.^6,7,49^ Sequence alignments with available protein sequences from the GenBank, SulfAtlas and PDB showed that the most similar protein with a known structure (46% sequence identity) is the HhSulf_S1_S16 gut microbial 6S-GalNAc sulfatase (PDB entry 6UST) from *Hungatella hathewayi*,^48^ while AMOR_S1_16A shares 35.6% sequence identity with the BT3769_S1_16 (PDB entry 7OZA) and 31% with the BT3057_S1_16 (PDB entry 7OZ9) G4*S*/4S-GalNAc sulfatases from *Bacteroides thetaiotaomicron*.^14^

According to Luis et al., the majority of sequences in the S1_16 subfamily are derived from marine environments.^14^ However, despite the prevalence of G4S and G6S in marine polysaccharides, no marine sulfatases from this subfamily have yet been demonstrated.

Already known carrageenan-active sulfatases belong to the S1 subfamilies 7, 17, 19, and 81.^6−8^ MSA of AMOR_S1_16A with known carrageenan sulfatases showed the highest sequence identity (34%) with PfS1_19B, an exoacting G4S sulfatase from *P. fuliginea*.^13^ MSA with identified sulfatases revealed the presence of conserved amino acid residues characteristic for the S1 sulfatases (Supplementary Figure S1). The sulfatase signature motif (in AMOR_S1_16A: ^58^CSPTRASILTGK^69^) is present in all the sulfatase sequences used in the alignment and contains the cysteine that is post-translationally modified into formyl-glycine, except for BT3057_S1_16 and BT3769_S1_16, which contains serine, often present in sulfatases from facultative or strictly anaerobic prokaryotes^6^ (Supplementary Figure S1). The amino acids involved in metal coordination are present in all the sulfatases (in AMOR_S1_16A: D18-D19-D263-N264) and are similar for all compared sulfatases except for the two S1_7 sulfatases where the asparagine is replaced by a histidine, which is seen in some cases^6,50^ (Supplementary Figure S1). A conserved tryptophan (W79 in AMOR_S1_16A) of S1_16 sulfatases is present in the S1_16 sequences and all the S1 sulfatases have the two polar residues Lys and His (K287 and H201 in AMOR_S1_16A) involved in substrate recognition (Supplementary Figure S1).

The gene encoding AMOR_S1_16A was codon optimized for expression in *E. coli* and recombinantly produced and purified with a C-terminal His_6_-tag and without the predicted N-terminal signal peptide (Supplementary Figure S2). Sulfatase activity was verified using the model substrate pNCS.

### Optimal Reaction Parameters

**Figure 2:**
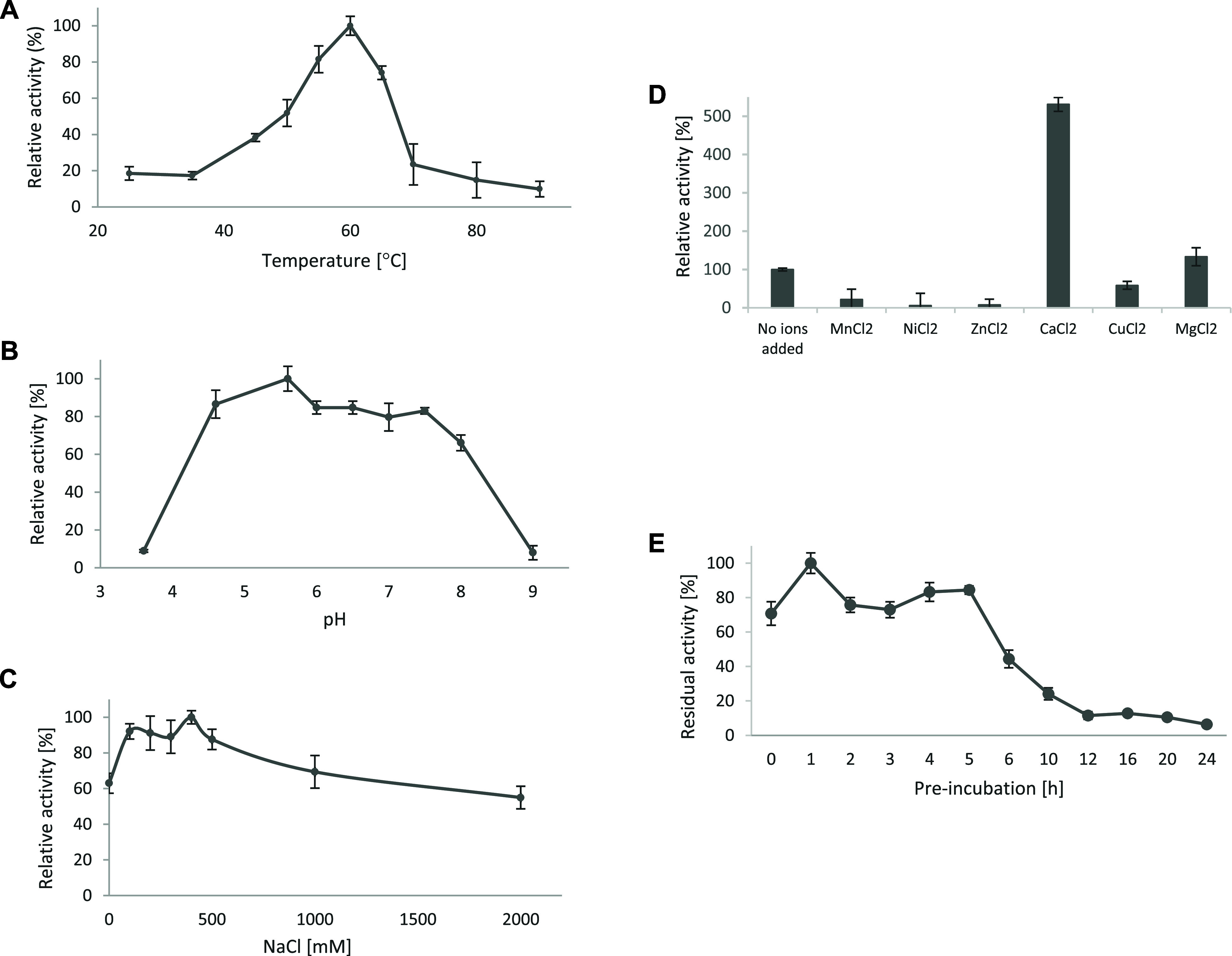
Activity characterization of AMOR_S1_16A. Effects of A: Temperature, B: pH, C: NaCl and D: Ions on relative enzyme activity. Panel E shows remaining activity after preincubation of AMOR_S1_16A for different times at 60 °C. All reaction mixtures were prepared using 1 μM AMOR_S1_16A and 2.5 mM pNCS and reaction products were measured after 40 min reaction. Experiments were performed in triplicates and results are shown as mean ± standard deviations. Activity was normalized to 100% for the condition with the highest product level. For the effects of cations, activity was normalized to 100% for the reaction with no ions added.

In 40 min reactions, AMOR_S1_16A exhibited the highest activity at 60 °C, while 19% of this maximum activity was maintained at 25 °C and 10% at 90 °C (Figure 2A). An optimal reaction temperature of 60 °C, is considerably higher than what has been determined for other carrageenan sulfatases, e.g., 34 °C for the endoacting 4S-ιota-carrageenan sulfatase from *Paraglaciecola atlantica* PaS1_19A, and 25 °C for the two endoacting κ-carrageenan sulfatases, Q15XH1_S1_7 and Q15XG7_S1_19 from *P. atlantica*.^7,12^

The high optimal reaction temperature for AMOR_S1_16A aligns well with the source of the enzyme, as the sequence was obtained from a marine genome collected from a chamber located in seawater at approximately 70 °C. Two alginate lyases, from the families PL7 (Genbank accession number MH727998) and PL17 (Genbank accession number MT444120), identified from the same source have optimal reaction temperatures at 65 °C (50 min reactions) and 90 °C (5 min reactions), respectively.^18,19^ A newly characterized κ-carrageenase has also shown to be heat-resistant, capable of tolerating reaction temperatures up to 100 °C.^51^ The effect of pH on enzyme activity was tested in the range of 3.6–9.0 at 60 °C. AMOR_S1_16A was active over a broad pH range with the highest activity on pNCS around pH 5.6 (Figure 2B), which is slightly lower compared to pH-optima reported for other carrageenan sulfatases.^11,17,52,53^ However, AMOR_S1_16A maintained more than 80% of its maximum activity in the pH range 4.6–7.5 (Figure 2B). AMOR_S1_16A was also shown to be active over a broad salinity range from 0 to 2000 mM NaCl, with the highest activity at approximately 400 mM NaCl, which is close to the NaCl concentration of seawater (Figure 2C).

The effects of divalent ions were determined in 25 mM NaOAc, pH 5.6, 400 mM NaCl, at 60 °C using 10 mM of MnCl_2_, NiCl_2_, ZnCl_2_, CaCl_2_, CuCl_2_, or MgCl_2_. Experiments testing different divalent ions showed that the presence of both Ca^2+^ and Mg^2+^ induced activity while Mn^2+^, Ni^2+^, and Cu^2+^ inhibited the activity of AMOR_S1_16A (Figure 2D). The presence of calcium ions induced activity by 430% compared to the reaction without added ions (Figure 2D). The calcium dependency of AMOR_S1_16A aligns well with the fact that S1 sulfatases contain a calcium binding site.^8,14^

The thermostability of AMOR_S1_16A was evaluated by measuring the sulfatase activity in a standard reaction after preincubation of the enzyme without substrate at 60 °C for 0–24 h (Figure 2E). AMOR_S1_16A maintained high activity upon incubation at 60 °C for up to around 5 h. Longer incubation resulted in a clear reduction of activity, with almost no activity remaining after 12 h of incubation (Figure 2E). Further experiments where the enzyme was incubated for 16 h at different temperatures showed that the enzyme was stable at 50 °C but lost all activity at 70 °C (data not shown). In comparison, the heat-resistant κ-carrageenase from the marine bacterium *Microbulbifer thermotolerans*, retained approximately 50% of its activity after 60 min of incubation at 100 °C,^51^ while a thermostable fucoidan sulfatase from a *Pseudoalteromonas* sp. retained almost 60% of its maximum activity after 12 h of incubation at its optimal reaction temperature of 68 °C. To our knowledge, no thermostable κ-carrageenan sulfatases have previously been described.

### Production of Oligosaccharide Substrates

To produce a variety of different substrates for enzyme activity tests, oligomers were generated by partial hydrolysis of native full-length commercial substrates using TFA (1 M TFA at 60 °C for 1 h). κ-carrageenan oligomers were additionally produced by hydrolyzing native full-length κ-carrageenan with the κ-carrageenase ZgCgk16A from *Z. galactanivorans* (EC number 3.2.1.83), which exclusively hydrolyzes the β-1,4 linkages, generating oligosaccharides with DA at the nonreducing end and G4S at the reducing end.^54,55^ The production of oligosaccharides was verified by SEC-RI and MALDI-TOF mass spectrometry (MS) (only shown and discussed for κ-carrageenan). Using SEC-RI, the molecular weight of native κ-carrageenan was estimated to be approximately 800 kDa (Supplementary Figures S3 and S4). Upon TFA treatment, the molecular weight was reduced to less than 6 kDa (Supplementary Figure S3). A similar reduction in size was observed after treating native full-length κ-carrageenan with ZgCgk16A (Supplementary Figure S3). Due to the carrageenan being a charged compound, it will exhibit a rod-like behavior in solution. Furthermore, the hydrodynamic radii will be larger due to counterions, resulting in an apparently higher molecular weight compared to the pullulan standard. Nevertheless, a clear degradation of the full-length native substrates was verified using SEC-RI, when comparing the polymers with the oligomers.

MALDI-TOF mass spectrometry analysis in the negative-ion mode confirmed that κ-carrageenan was hydrolyzed into a blend of sulfated oligosaccharides with varying degrees of polymerization (DP) and sulfation (Supplementary Table S2). The most abundant oligosaccharide detected in the enzymatic hydrolysate of κ-carrageenan was the tetramer κ-neocarratetraose, followed by shorter units (DP3, DP2, and G4S). Larger oligosaccharides, up to DP16 of approximately 3 kDa, were detected in lower amounts (Supplementary Table S2). Our identification of κ-neocarratetraose as the main component of the κ-carrageenan enzymatic hydrolysate is in consistence with characterization studies of ZgCgk16A, which show that κ-neocarratetraose is the main end product formed by this enzyme.^54,55^ In contrast to the enzymatic hydrolysate, analysis of TFA-hydrolyzed κ-carrageenan revealed a more heterogeneous mixture of products and a much larger abundance of oligosaccharides with an odd number of DP (DP5, DP7, DP9, DP11, DP13, DP15) (Supplementary Table S2). Interestingly, most of these oligosaccharides have a G4S unit at both the nonreducing and reducing ends. This can be explained by the instability of DA units at the reducing end under acidic conditions, meaning that the DA unit will be lost and the oligosaccharide will become one unit shorter with G4S at the reducing end.^56^ This also means that only odd-numbered oligosaccharides will have a G4S at the nonreducing end. Similar results have been reported for the mild acid hydrolysis of κ-carrageenan.^56,57^

Previous studies using MALDI-TOF mass spectrometry have shown that the ionization efficiency of carrageenan oligosaccharides drops as the molecular weight increases^38,58^ indicating that larger fragments may have gone undetected.

Dehydration products and loss of sulfite groups (which translates into a functional loss of a sulfate group), are common reactions occurring during MALDI-TOF analysis of sulfated oligosaccharides.^58,59^ Such in-source substrate modifications were detected in our analyses and support the assignment of annotations in the enzymatic hydrolysate (Supplementary Table S2). However, a lack of discrimination power between desulfation reactions caused by TFA or sulfatase treatment, or subsequently during MALDI-TOF analysis, represents a major challenge in the application of this technique to analyze sulfatase activity in TFA-hydrolyzed κ-carrageenan. For this reason, we turned to HPAEC as screening method to monitor the release of sulfate ions from tested substrates upon incubation with AMOR_S1_16A.

### Substrate Specificity

To determine the substrate specificity of AMOR_S1_16A, native polymeric substrates, oligomers, and monomers were used for activity assays. Using HPAEC for detection of hydrolyzed sulfate ions, it was shown that AMOR_S1_16A has sulfatase activity on TFA-hydrolyzed κ-carrageenan (Supplementary Figure S5), while no sulfate release was detected in reactions with native polymeric κ-carrageenan nor commercial intact and TFA-hydrolyzed agar, ι-carrageenan, λ-carrageenan, or any of the fucoidan substrates tested in this study. These results indicate that AMOR_S1_16A is a 4-O sulfatase acting on κ-carrageenan oligomers.

Like κ-carrageenan, ι-carrageenan contains G4S, but in ι-carrageenan, the G4S is located between two sulfated DA units, while in κ-carrageenan, the G4S is located between two neutral DA units. This, i.e., the nature of neighboring sugars with or without sulfate substitutions, may explain why AMOR_S1_16A is only active on κ-carrageenan. Analysis of substrate specificity of the two κ-carrageenan sulfatases Q15XH1_S1_7 and Q15XG7_S1_19 from *P. atlantica* showed that these enzymes also only act on κ-carrageenan and not on ι-carrageenan.^12^

Interestingly, AMOR_S1_16A showed no sulfate release in reactions with κ-carrageenan oligomers produced by reaction with the κ-carrageenase ZgCgk16A. This finding is intriguing since current models of carrageenan catabolism pathways suggest that sulfatases act subsequent to carrageenases.^5,13^ κ-carrageenan is composed of repeating units of G4S and DA that are bound together by alternating α- and β-bonds. Carrageenases, such as ZgCgk16A, target the internal β-bonds between G4S and DA, yielding κ-carrageenan oligomers having DA at the nonreducing end and an α-linked G4S at the reducing end^36,54^ (Supplementary Figure S6). The acid hydrolysis is not controlled like the enzymatic hydrolysis, and the product is thus expected to be a mixture of oligomers with different sugars at the nonreducing and reducing ends (Supplementary Figure S6). This is in accordance with the MALDI-TOF data, which additionally show a higher abundance of odd-numbered oligomers having G4S at the nonreducing end, probably due to the instability of DA in acid^56^ (Supplementary Table S2). Considering that the chemical and enzymatic routes produce different oligomers led to speculations about the importance of the sugar residues at the ends, which was further investigated by NMR spectroscopy.

AMOR_S1_16A belongs to a subfamily with reported activity on G4S and G6S. AMOR_S1_16A was tested for activity on commercial G6S, but no sulfate release was observed (data not shown). Reported 4-O sulfatases with activities on carrageenans belong to the S1 subfamilies 7, 17, 19, and 81.^5,11−13,60^ Two S1_16 sulfatases, BT3057 and BT3796, from the human gut bacterium *B. thetaiotaomicron*, are active on G4S and 4S-GalNAc from colonic mucin and not on carrageenan.^14,61^ Therefore, AMOR_S1_16A was additionally tested for activity on the G4S and the 4S-GalNAc monosaccharides. While some sulfate release was observed for the G4S substrate, no sulfate release was observed when reacting AMOR_S1_16A with 4S-GalNAc (results not shown). To our knowledge, this is the first proof of a bacterial sulfatase active on G4S of κ-carrageenan. Thus, AMOR_S1_16A represents a new activity within the S1_16 subfamily, i.e., κ-carrageenan oligomers.

It should be noted that the GHs found on the contig from which AMOR_S1_16A was derived include, among others, putative fucosidases and galactosidases, which may play a role in processing seaweed polysaccharides like fucoidan. Considering the activity of AMOR_S1_16A, it is intriguing that enzymes putatively involved in carrageenan metabolism are lacking. It is conceivable that the AMOR_S1_16A has additional substrates that remain to be discovered, for example certain fucoidan types not tested in this study, or that the GHs on the same contig have hitherto unknown activities related to carrageenan metabolism.

### Mode of Action

**Figure 3:**
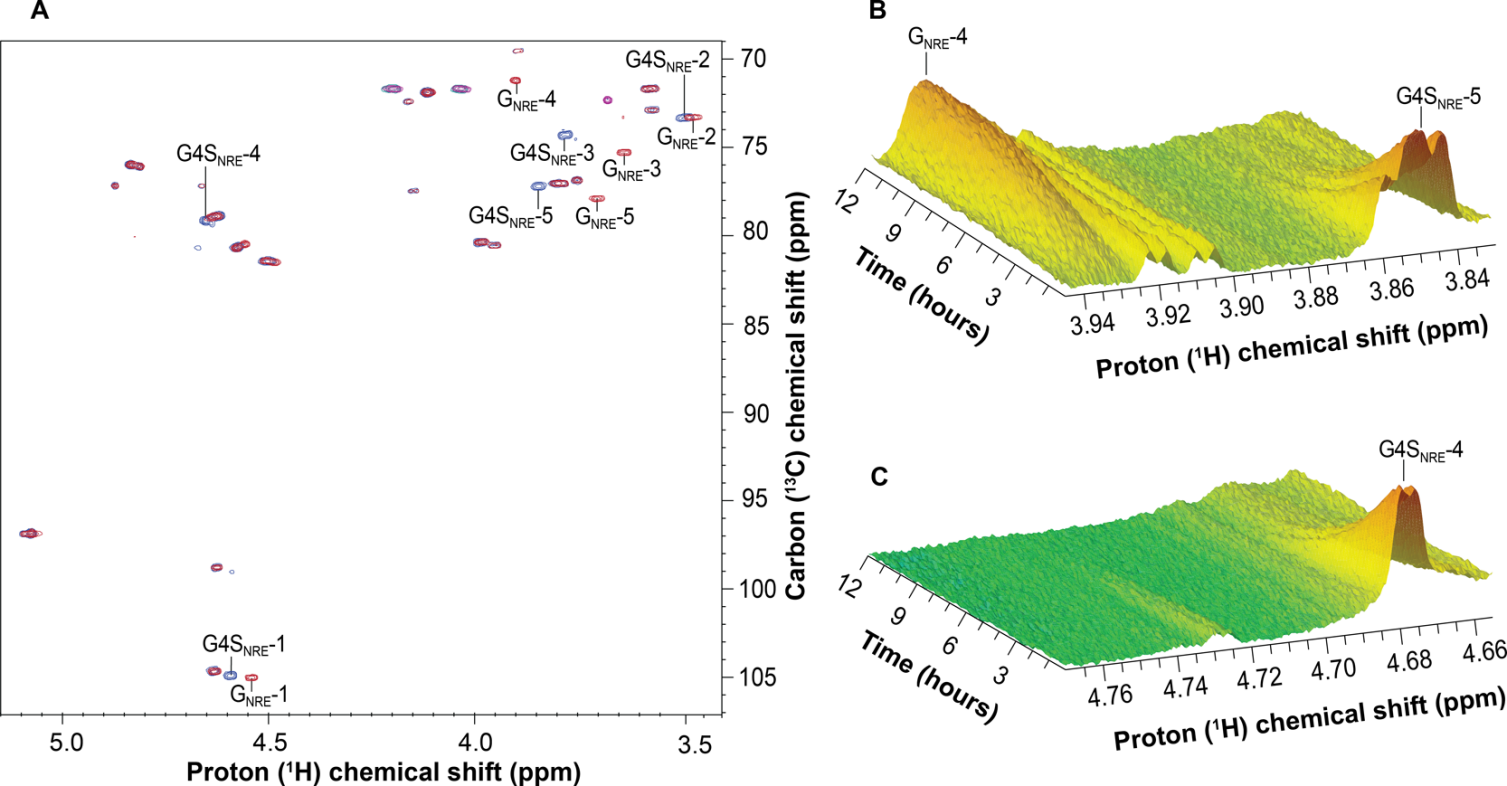
NMR spectra show a complete desulfation of the Gal4SNRE in TFA-hydrolyzed κ-carrageenan in the presence of AMOR_S1_16A. (A) Overlay of HSQC spectra that show desulfation of κ-carrageenan. Spectrum of untreated substrate (blue/green) compared to the spectrum recorded after reaction with 10 μM AMOR_S1_16A (red/pink) shows desulfation of the G4S residue at the nonreducing ends (annotated peaks). Correlations in the HSQC spectra indicate chemical shifts of protons and carbons that are directly bonded. (B,C) ^1^H-monitored time-resolved spectrum showing decrease in G4S nonreducing end signals (H-4:4.68 ppm and H-5:3.85 ppm) and increase in G nonreducing end signal (H-4:3.93 ppm). Reaction mixture comprised 10 mg/mL κ-carrageenan (TFA-hydrolyzed) in 10 mM NaOAc, 200 mM NaCl, 10 mM CaCl_2_, pH 5.6 in 99.9% D_2_O and 32 μM of AMOR_S1_16A. G4S: β-d-galactopyranose 4-sulfate, G: β-d-galactopyranose, NRE: nonreducing end. Numbers (1–5) indicated proton/carbon position within each residue.

The activity of AMOR_S1_16A was assessed using NMR spectroscopy to provide additional insight into the specific sulfate group targeted by the enzyme and to discriminate between an endo- versus exomode of action. Using 2D NMR spectroscopy, a full assignment of the TFA-hydrolyzed κ-carrageenan both before and after treatment with AMOR_S1_16A was completed (Supplementary Figure S7). The NMR analysis revealed κ-carrageenan oligomers with G4S residues at the nonreducing end (Supplementary Figure S7), consistent with the MALDI-TOF mass spectrometry data (Supplementary Table S2) and literature on acid hydrolysis of κ-carrageenan.^56,57^ After treatment with AMOR_S1_16A, signals associated with the G4S nonreducing end residue (G4S_NRE_) disappeared and were replaced by a nonsulfated galactose at the nonreducing end (G_NRE_) (Supplementary Figure S7), while the internal G4S signals (G4S) and those associated with the reducing end (G4S_RE_) remained (Supplementary Figure S7). When monitoring the reaction in real-time, the H-4 (4.68 ppm) and H-5 (3.85 ppm) protons of the G4S at the nonreducing end (G4S_NRE_-4 and G4S_NRE_-5) decreased, while at the same time the H-4 (3.93 ppm) of the nonsulfated galactose nonreducing end residue (G_NRE_) increased (Figure 3A–C). This provides evidence that AMOR_S1_16A has an exomode of action, desulfating only the nonreducing end residue of κ-carrageenan oligomers. Moreover, using HPAEC for detection of released sulfate ions (Supplementary Figure S5), it was shown that 0.04 mg/mL sulfate was released from 1 mg/mL κ-carrageenan hydrolysate. κ-carrageenan has a sulfate content around 25% (w/w), corresponding to 0.25 mg/mL sulfate. Theoretically, random acid hydrolysis will result in oligosaccharides where only 50% have a sulfate group at the nonreducing end. If we use an average DP of 5 for these oligosaccharides, with 33% of the sulfate at nonreducing end, we can estimate the maximum sulfate release by the enzyme to be 0.25 mg/mL × 0.5 × 0.33 = 0.04 mg/mL. This indicates complete desulfation of the G4S at the nonreducing end by the enzyme. The fact that AMOR_S1_16A can only desulfate the nonreducing end residue of κ-carrageenan would explain why AMOR_S1_16A does not release sulfates from the κ-carrageenan oligomers produced by the κ-carrageenase ZgCgk16A. As stated above, carrageenases specifically cleave the internal β-linkages between G4S and DA in the backbone of the carrageenan chains, leaving DA-G4S at the nonreducing ends.^54^ As observed by NMR (Supplementary Figure S7) and MALDI-TOF mass spectroscopy (Supplementary Table S2), the TFA-hydrolyzed κ-carrageenan contains oligomers with G4S at the nonreducing ends. To produce oligomers with a nonreducing end G4S enzymatically, the action of an anhydro-galactosidase breaking the α-linkages between DA and G4S would be needed. A few carrageenan α-galactosidases have been described, but as far as we know, the described enzymes react on desulfated carrageenan substrates.^5,13^ Hence, the proposed carrageenan degradation pathway described for *Z. galactanivorans*(5) and *Pseudoalteromonas carrageenovora*,^13^ where carrageenases initiate the degradation followed by sulfatases and then α-galactosidases, might not be universal for all marine bacteria and/or some enzyme functions are yet to be discovered.^62^ This is indeed the case for *Pseudoalteromonas haloplanktis*, where a recent discovery shows that sulfatases initiate the carrageenan catabolism followed by action of carrageenases.^63^

A galactosidase producing the right substrate for AMOR_S1_16A might exist in some of the genes located on the same contig as AMOR_S1_16A, but that is only speculations and should be tested experimentally. However, this is beyond the scope of this study.

### Crystal Structure of AMOR_S1_16A

**Figure 4:**
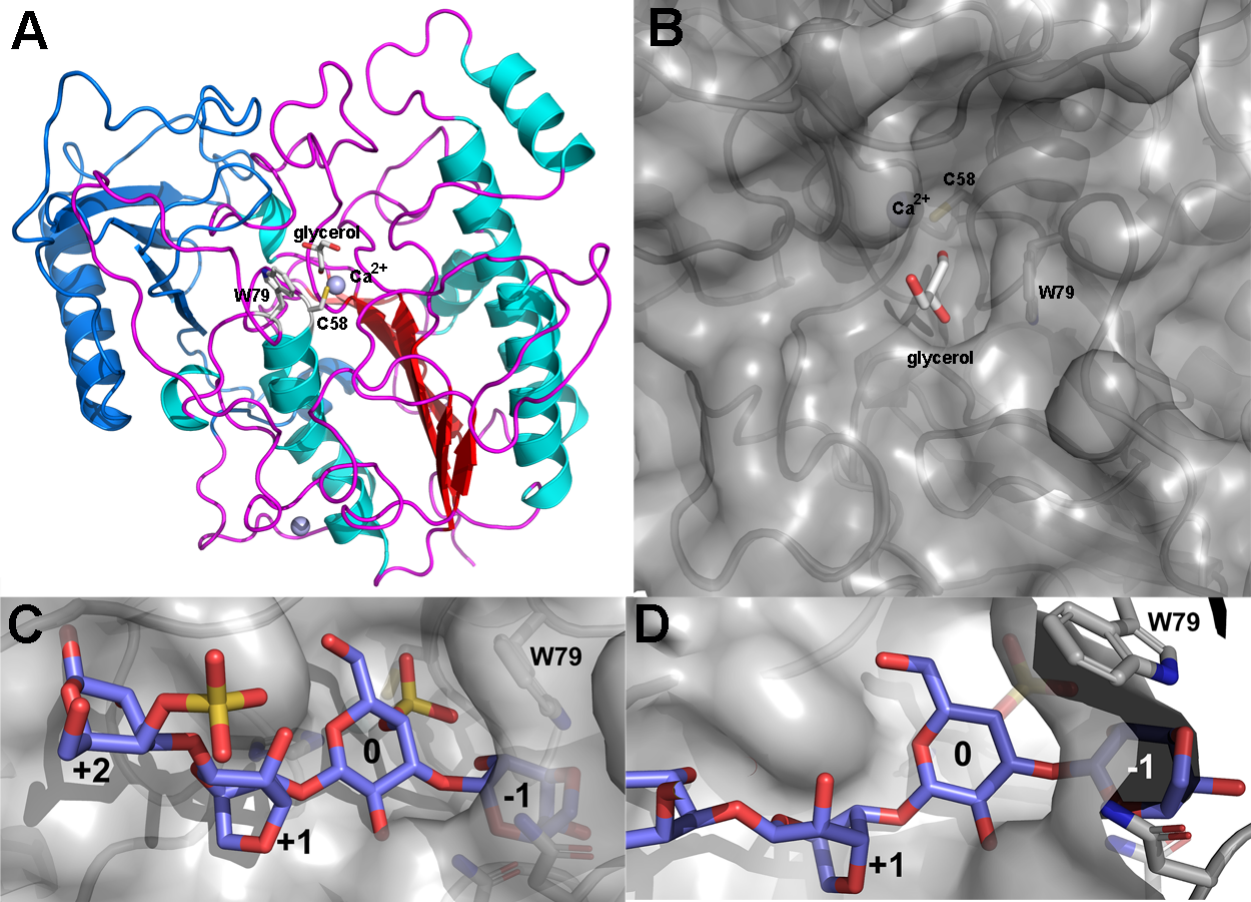
Crystal structure representations of AMOR_S1_16A. (A) Global cartoon representation of the overall fold; the structural elements of the N-terminal α/β/α-fold are colored red (b-strands), cyan (a-helices), and magenta (loops/connecting regions), respectively, and the C-terminal domain, present in all S1 sulfatases, is colored in blue. Positions of the catalytic relevant Ca^2+^ ion and cysteine are labeled, as well as a glycerol trapped in the active site pocket and a tryptophan (W79) that forms a wall of the active site pocket. (B) Surface overview of active site pocket with a glycerol entrapped and the tryptophan (W79) blocking at the end. (C,D) Docking of a k-carrageenan tetrasaccharide into the active site evidence the fact that the presence of this tryptophan blocks the pocket at the nonreducing end, and this steric hindrance leads to the absence of a −S1 sub-binding site in AMOR_S1_16A.

To provide insights into the molecular basis of AMOR_S1_16A's activity and residues guiding substrate specificity, we determined the X-ray crystal structure of AMOR_S1_16A at 3.1 Å resolution by molecular replacement using the model predicted by Alphafold2^43^ as starting point. The overall structure has the classical S1 sulfatase 3D arrangement composed of an N-terminal α/β/α-fold with a small C-terminal subdomain (Figure 4A), and the active site is located in a central pocket (Figure 4B) that is blocked off on one side by a conserved tryptophan residue (Trp79) (Figure 4C,D). The plane of this tryptophan acts as a sort of a barrier, possibly stacking against the sugar-plane of the unit bound in the S0 site, the region accommodating the carbohydrate portion of the sulfated residue (for subsite nomenclature see Hettle et al.^60^) (Figure 4C,D). This tryptophan has been shown to be crucial for 4-O specificity/activity in BT3796_S1_16 and BT3057_S1_16 (7OZA, 7OZ9).^14^

Molecular docking of a κ-carrageenan tetrasaccharide into the active site of AMOR_S1_16A indicates that the presence of Trp79 blocks the pocket at the nonreducing end, and this steric hindrance leads to the absence of a −S1 sub-binding site in AMOR_S1_16A (Figure 4C,D). Consequently, the active site pocket is compatible with AMOR_S1_16A being active on the G4S at the nonreducing end, leaving room only for S0 and S + 1 binding sites. This is in contrast to other κ-carrageenan active 4-O sulfatases: for example, the PfS1_19b exoacting sulfatase interacts with a disaccharide at the nonreducing end of a κ-carrageenan; thus, the S-subsite is not on the terminal residue. Rather, the 0-subsite is found one residue away from the nonreducing end.^13^

**Figure 5:**
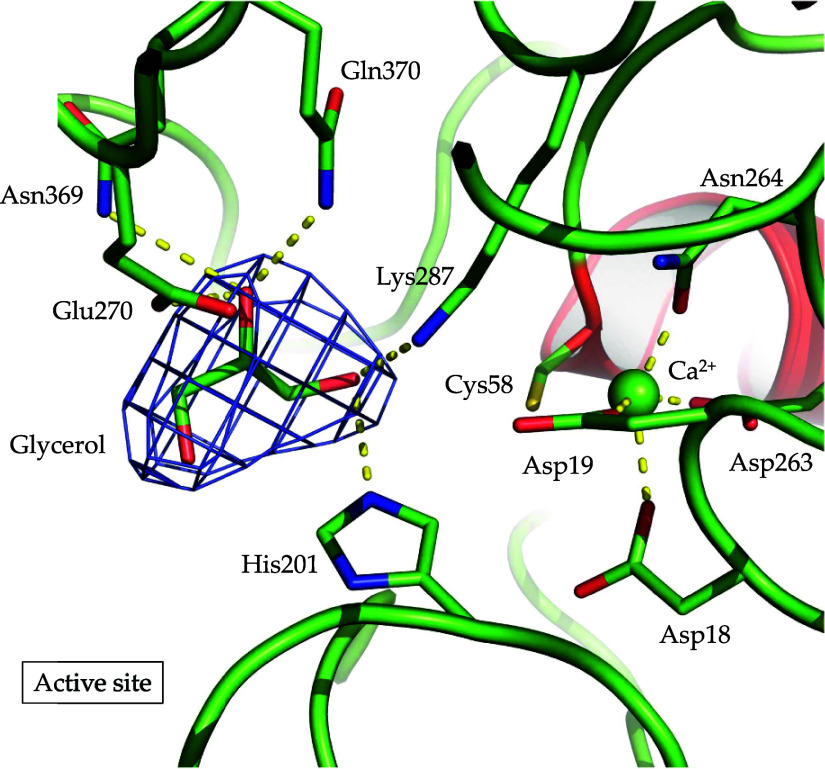
Ribbon representation of the active site of AMOR_S1_16A. Residues coordinating the solvent molecule glycerol and the Ca^2+^ ion, which are located at the active site of AMOR_S1_16A are labeled.

The active site of AMOR_S1_16A revealed the presence of a Ca^2+^ ion, which is conserved in all known S1 sulfatases with 3D structures so far (Figure 5). Strictly conserved residues involved in Ca^2+^ coordination are Asp18, Asp19, Asp263, and Asn264 (Figure 5). The crystal structure of AMOR_S1_16A showed no evidence of the catalytic nucleophile formylglycine residue, but instead the unmodified Cys58 (Figure 5). AMOR_S1_16A was produced without coexpression of the formylglycine generating enzyme, and it seems that the sulfatase maturation system present in *E. coli* was able to modify enough cysteines for activity but not enough to be seen in the crystal structure. This has been observed for several S1 sulfatase crystal structures, e.g., the ι-carrageenan sulfatase PsS1_19A (PDB entry 6BIA)^60^ and the fucoidan-active sulfatase PsFucS1 (PDB entry 7AJ0).^15^

The active site binds a glycerol molecule from the cryoprotectant, involving the amino acids Lys287, His201, Glu270, Gln370 and Asn369 (Figure 5). While the first two residues (Lys287 and His201) belong to the polar residues that are conserved throughout all S1 sulfatases and are involved in binding the sulfate ester that is cleaved,^14^ Glu270, Asn369 and Gln370 are positioned to recognize the saccharide unit at the S0 binding site that bears the sulfate ester. These residues are less conserved and vary among the different S1_16 enzymes for which structures have been determined.

**Figure 6:**
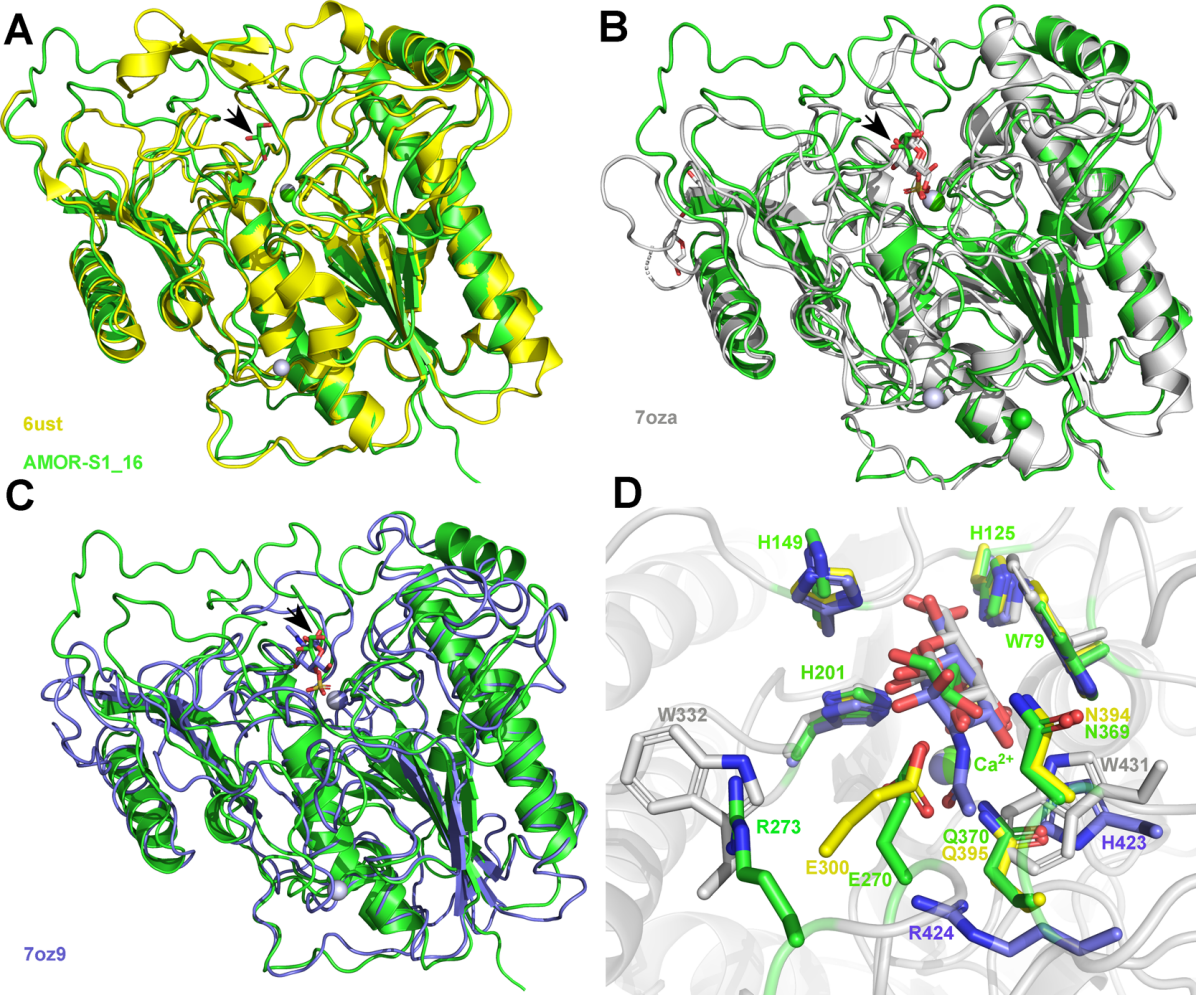
Structural comparison to crystal structures of S1_16 sulfatases. Cartoon representation of the overall superimposition of crystal structures of AMOR_S1_16, colored green, with (A) 6UST, colored yellow, (B) 7OZA, colored gray, and (C) 7OZ9, colored pale blue, highlighting the similarity of the core structure, while the loop regions surrounding the ligand binding site are more variable. In all three panels black arrows indicate the ligand positions in the active site pocket. Respective ligands are drawn as sticks. These ligands are zoomed on and superimposed in panel 5D. (D) Zoom into the active site pocket overlaying the different ligands present in the 3D crystal structures. Colors are the same as in panels A–C. Orientation in panel D is turned by 180° clock-wise with respect to that presented in panels A–C. Strictly conserved residues of all four structures, namely Trp79, His125, His149, His201, and the Ca^2+^ are only labeled in green for AMOR_S1_16, while diverging residues close to the S0 and +S1 binding sites are labeled in their respective colors as in panel A–C.

Structural comparisons of AMOR_S1_16A and the 6S-GalNAc sulfatase from *H. hathewayi* (6UST^48^; Figure 6A) and the 4S-Gal/GalNAc sulfatases BT3796_S1_16 (7OZA; Figure 6B) and BT3057-S1_16 (7OZ9; Figure 6C) from *B. thetaiotaomicron*,^14^ show that AMOR_S1_16A is highly similar to some of the few S1_16 solved structures (rmsd values of Cα superimposition are given in Supplementary Table S4). Besides the Trp (Trp79 in AMOR_S1_16A) that are blocking the active site pocket, two histidine residues, His125 and His149 in AMOR_S1_16, in the vicinity of the substrate sugar-unit bound at S0 are also well conserved and characteristic of S1_16 sulfatases (Figure 6D, Supplementary Figure S8). The main differences are localized at the C-terminal end and in the specific loop (266–276 in AMOR_S1_16A) near to the active site pocket. Unfortunately, we did not manage to get a crystal in complex with κ-carrageenan, but Glu270, Asn369, and Gln370 seem to be positioned close to the O2 position of the G4S sugar unit that has to be bound in the S0-subsite, leaving no room for an acetyl group (Figure 6D, Supplementary Figure S8). This is in contrast to what is seen in 7OZA and 7OZ9, where His423 and Arg424 (in 7OZ9) and Trp431 (in 7OZA) allow for the presence of a GalNAc unit (Figure 6D, Supplementary Figure S8). The closest relative 6UST possesses the same residue configuration at these positions (Figure 6D, Supplementary Figure S8), but with the positioning of the Glu in 6UST that differs from that of AMOR_S1_16A. Arg273 in AMOR_S1_16A seems possibly important for defining specificity of the positive sub-binding site + S1 in AMOR_S1_16A (Figure 6D, Supplementary Figure S8).

## Supporting Information Available

Multiple sequence alignment of selected S1 sulfatases; SDS-PAGE of purified AMOR_S1_16; size-exclusion chromatogram of native κ-carrageenan, TFA-hydrolyzed κ-carrageenan, and enzymatically hydrolyzed κ-carrageenan; size-exclusion chromatograms of pullulan standards; HPAEC chromatogram showing sulfate release when reacting AMOR_S1_16A with TFA-hydrolyzed κ-carrageenan; illustration of breakdown routes of κ-carrageenan using either GH16 κ-carrageenase or acid hydrolysis; ^1^H NMR spectra for enzymatic reaction and control sample; representation of active site residues in superimposed crystal structures of S1_16 sulfatases; X-ray crystallography data collection and refinement statistics; MALDI-TOFF mass spectroscopy data of the κ-carrageenan samples; ^1^H and ^13^C chemical shifts for enzymatic reaction of AMOR_S1_16A with TFA-hydrolyzed κ-carrageenan; and structural superimposition data of AMOR_S1_16A to the coordinates of the S1 sulfatases 6UST, 7OZA, and 7OZ9 (PDF)

## Abbreviations Used

4S-GalNAc: *N*-acetylgalactosamine-4-sulfate
6S-GalNAc: *N*-acetylgalactosamine-6-sulfate
AMOR: Arctic Mid-Ocean Ridges
DA: 3,6-anhydrogalactose
DP: degrees of polymerization
G: galactose
G4S: galactose-4-sulfate
G6S: galactose-6-sulfate
GH: glycosyl hydrolases
HPAEC: high performance anion exchange chromatography
MALDI-TOF: Matrix Assisted Laser Desorption Ionization-Time of Flight
MSA: multiple sequence alignment
NMR: nuclear magnetic resonance
pNCS: 4-nitrocatechol sulfate dipotassium salt
SEC-RI: size-exclusion chromatography
