# Supplementary material for: Identification and Characterization of a New Thermophilic κ-Carrageenan Sulfatase
Source: J Agric Food Chem. 2025 Jan 11;73(3):2044–55. doi: 10.1021/acs.jafc.4c09751 (PMC11760155; doi:10.1021/acs.jafc.4c09751)
Supplement: Supplementary file 1 — jf4c09751_si_001.pdf [file jf4c09751_si_001.pdf]

Supporting Information

Identification and Characterization of a New Thermophilic κ-carrageenan Sulfatase

Nanna Rhein-Knudsen*[1,2)], Diego S. Reyes-Weiss[1)], Leesa J. Klau[3,4)], Alexandra Jeudy[2)], Thomas Roret[2)], Runar Stokke[5)], Vincent G. H. Eijsink[1)], Finn L. Aachmann[3], Mirjam Czjzek[2)] and Svein Jarle Horn*[1)]

1) Faculty of Chemistry, Biotechnology, and Food Science, NMBU Norwegian University of Life Sciences, P.O. Box 5003, 1432 Aas, Norway

2) Sorbonne Universitée, CNRS, Integrative Biology of Marine Models, Station Biologique de Roscoff, 29680 Roscoff, France

3) Department of Biotechnology and Food Science, NTNU Norwegian University of Science and Technology, Sem Sælands vei 6/8,7419 Trondheim, Norway

4) Department of Process Technology, SINTEF Industry, Forskningsveien 1, 0373 Oslo, Norway

5) Department of Biological Sciences and Centre for Deep Sea Research, University of Bergen, 5020 Bergen, Norway

*Corresponding authors: Nanna Rhein-Knudsen nanna.rhein-knudsen@sb-roscoff.fr; Svein J. Horn svein.horn@nmbu.no

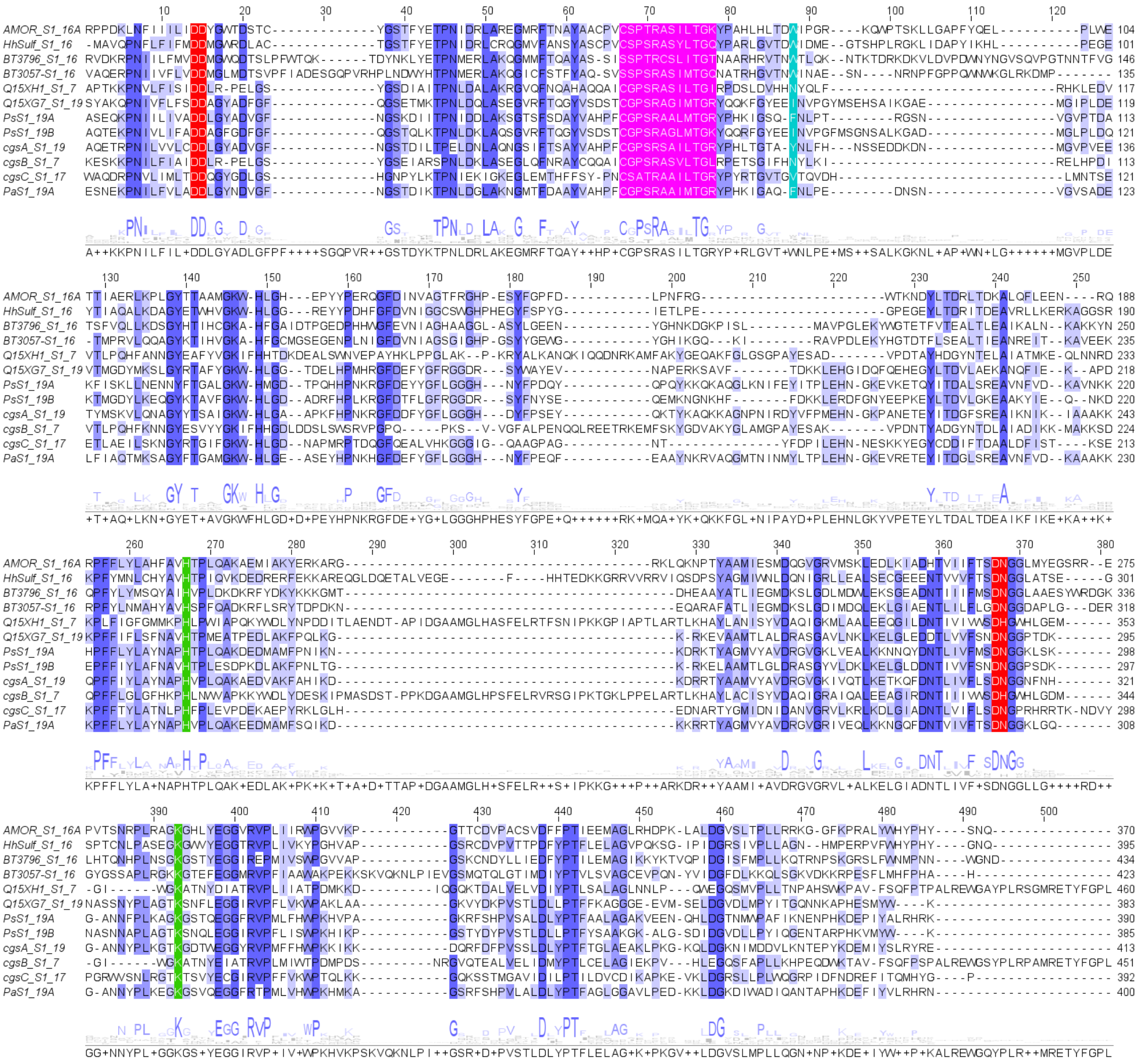

Figure S1. Multiple sequence alignment of selected S1 sulfatases. The sequences include: AMOR_S1_16A (GenBank accession number PP524981, PDB entry 9FO1), The *N*-acetylgalactosamine 6-sulfatase HhSulf_S1_16 (UniProt A0A174CV66, PDB entry: 6UST) from *Hungatella hathewayi*, the two galactose/*N*-acetylgalactosamine 4-sulfatases BT3796_S1_16 (UniProt Q8A171, PDB entry 7OZA) and BT3057_S1_16 (UniProt Q8A397, PDB entry 7OZ9) from *Bacteroides thetaiotaomicron* the two κ-carrageenan endo-acting galactose 4-sulfatases Q15XH1_S1_7 (Uniprot A0A9J9B7Z5) and Q15XG7_S1_19 (UniProt A0A9J9ESA2) from *Pseudoalteromonas atlantica,* the κ/ι-carrageenan endo-acting galactose 4-sulfatase PfS1_19A (UniProt A0A2S1KL01, PDB entry 6BIA) and the κ-carrageenan exo-acting galactose 4-sulfatase PfS1_19B (UniProt A0A833AJ03, PDB entry 6PRM) from *Pseudoalteromonas fuliginea,* the ι-carrageenan endo-acting galactose 4-sulfatase cgsA_S1_19 (UniProt G0L000), the κ-carrageenan endo-acting galactose 4-sulfatase cgsB_S1_7 (UniProt G0L7B4), and the α-

carrageenan 3,6-anhydro-galactose 2-sulfatase cgsC_S1_17 (UniProt G0L7B6) from *Zobellia galactanivorans,* and the ι-carrageenan endo-acting galactose 4-sulfatase PaS1_19A (UniProt A0A9J9B832) from *P. atlantica.* The S1 sulfatase signature motif is highlighted in pink, amino acids involved in metal coordination are highlighted in red, the conserved S1_16 tryptophan in turquoise, and conserved polar residues with known roles in substrate binding are highlighted in green.

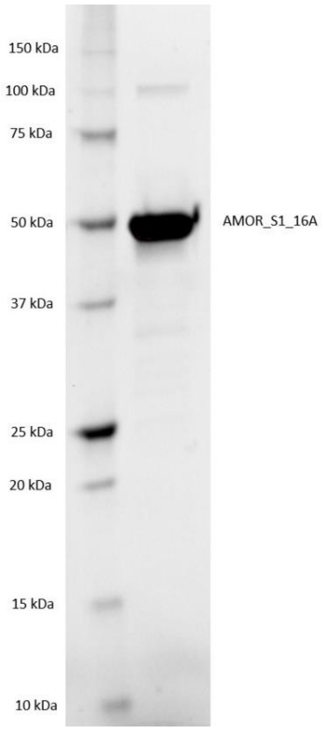

Figure S2. SDS-PAGE of purified AMOR_S1_16. AMOR_S1_16A was purified with a Ni^{2+} affinity HisTrap FF 5 mL column (GE HealthCare, Chicago, USA) following buffer-exchange using 25 mM NaOAc, pH 5.6, 200 mM NaCl and a HiPrep™ 26/10 desalting column (Cytiva, Sweden). Fist lane shows the Precision Plus Protein Marker (BioRad) and second lane the purified AMOR_S1_16A (52.9 kDa).

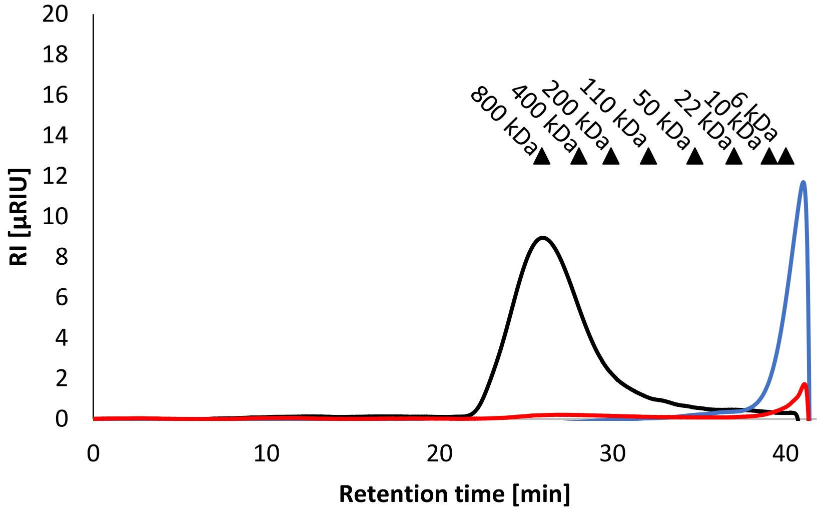

Figure S3. Size exclusion chromatogram (SEC-RI) for native κ-carrageenan (black), TFA-hydrolyzed κ-carrageenan (blue), and enzymatically hydrolyzed κ-carrageenan (red). The elution positions and sizes of pullulan standards are indicated in the chromatogram as black triangles (see also Figure S4). All samples were analyzed on an Ultimate3000 system using a TSKgel®PWXL guard column connected in series to a TSKgel®G4000PWXL column and a TSKgel®G5000PWXL column. Elution was performed with 0.15 M $NaNO_3$, 0.01 M EDTA, pH 6.0, at a flowrate of 0.5 mL/min.

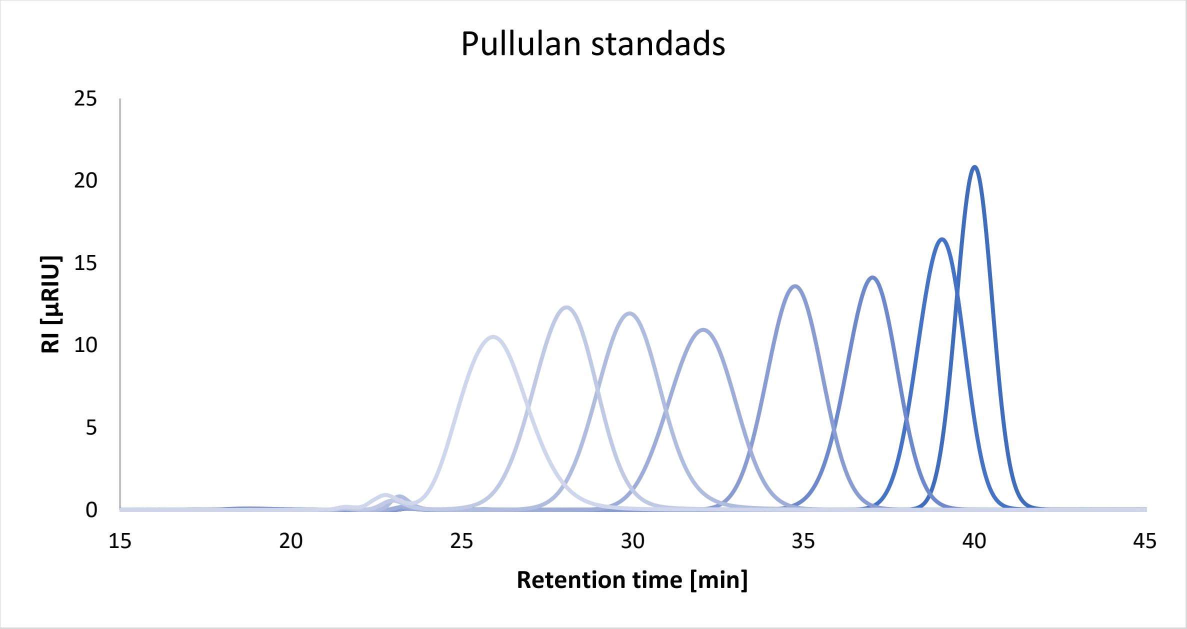

Figure S4. Size-exclusion chromatograms of pullulan standards shown in a gradient of blue tones and in order of elution: 800 kDa, 400 kDa, 200 kDa, 110 kDa, 50 kDa, 22 kDa, 10 kDa, and 6 kDa.

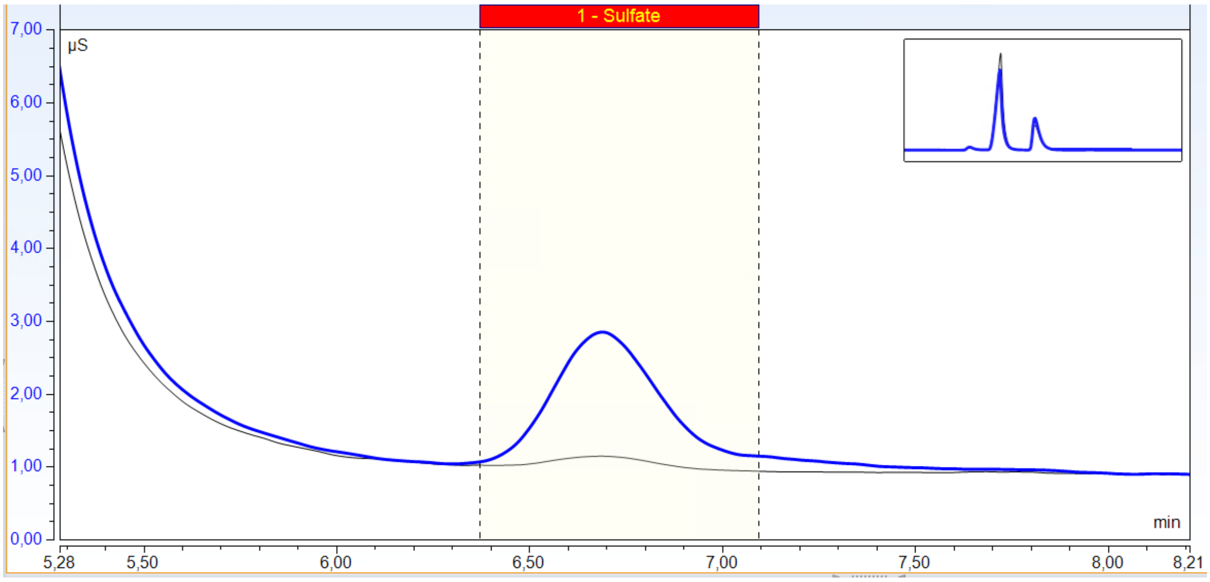

Figure S5. High performance anion exchange (HPAEC) chromatogram for detection of free sulfate ions of TFA-hydrolyzed κ-carrageenan (black) and reaction of AMOR_S1_16A with TFA-hydrolyzed κ-carrageenan (1 mg/mL) under optimal reaction conditions (25 mM NaOAc, pH 5.6, 400 mM NaCl, 20 mM $CaCl_2$ at 60 °C) overnight (blue). The ions were separated on an AS-11-HC anion-exchange column with accompanying AG11-HC guard column using 5 mM KOH. Sulfate peak identification was done using K_2SO_4 and sulfate retention time is highlighted in red. The peak corresponds to a sulfate concentration of 0.04 mg/mL. The inset shows the whole chromatogram with the buffer peaks.

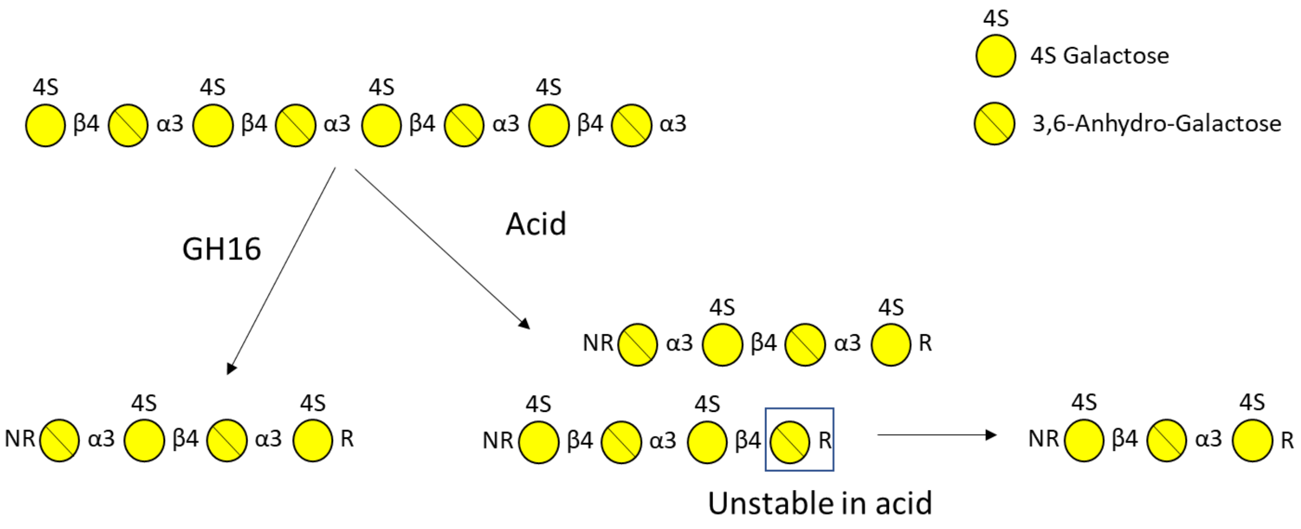

Figure S6. Illustration of breakdown routes of κ-carrageenan using either GH16 κ-carrageenases or acid hydrolysis. Note that enzyme hydrolysis, using carrageenase, exclusively yields oligosaccharides with an anhydro-galactose at the non-reducing end. N=reducing end. NR= non-reducing end.

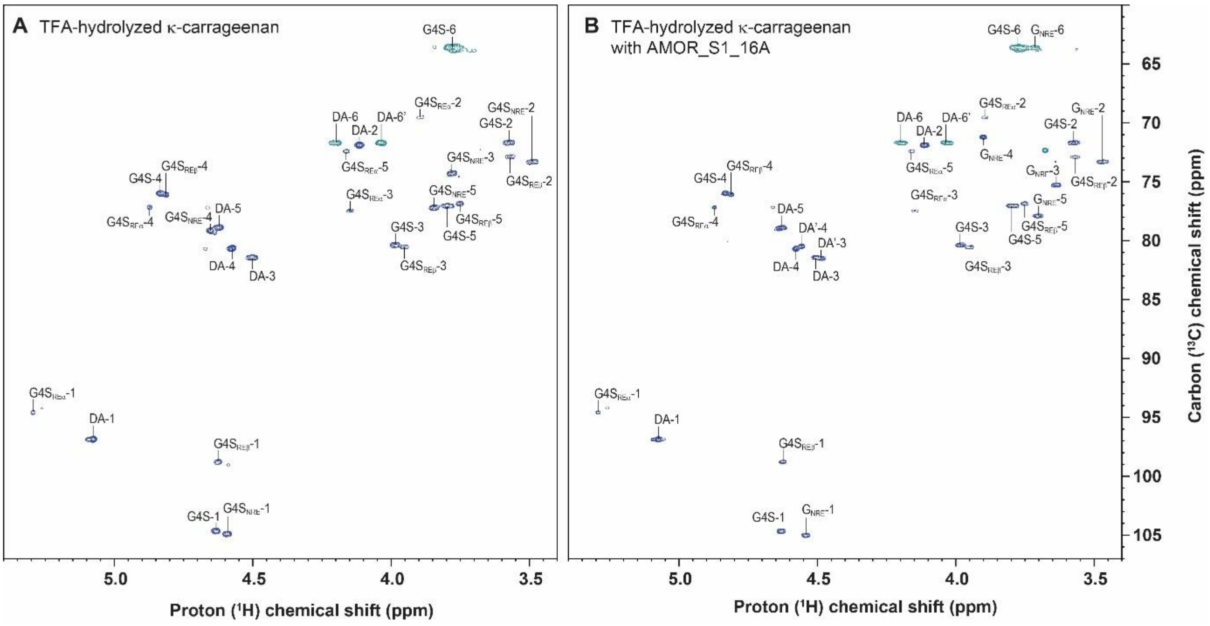

Figure S7. Annotated HSQC spectra for A) TFA-hydrolyzed κ-carrageenan and B) TFA-hydrolyzed κ-carrageenan treated with AMOR_S1_16A (10 µM AMOR_S1_16 and 2 mg/mL substrate under optimal reaction conditions). Spectra acquired in 99.9% D_2O at 25 ^oC. Correlations indicate chemical shifts of protons and carbons that are directly bonded. DA: 4-linked 3,6-anhydro-α-D-galactopyranose, G4S: 3-linked β-D-galactopyranose 4-sulfate, G: β-D-galactopyranose, NRE: non-reducing end, REα: alpha reducing end, REβ: beta reducing end. Numbers (1-6) indicate proton/carbon position within each residue.

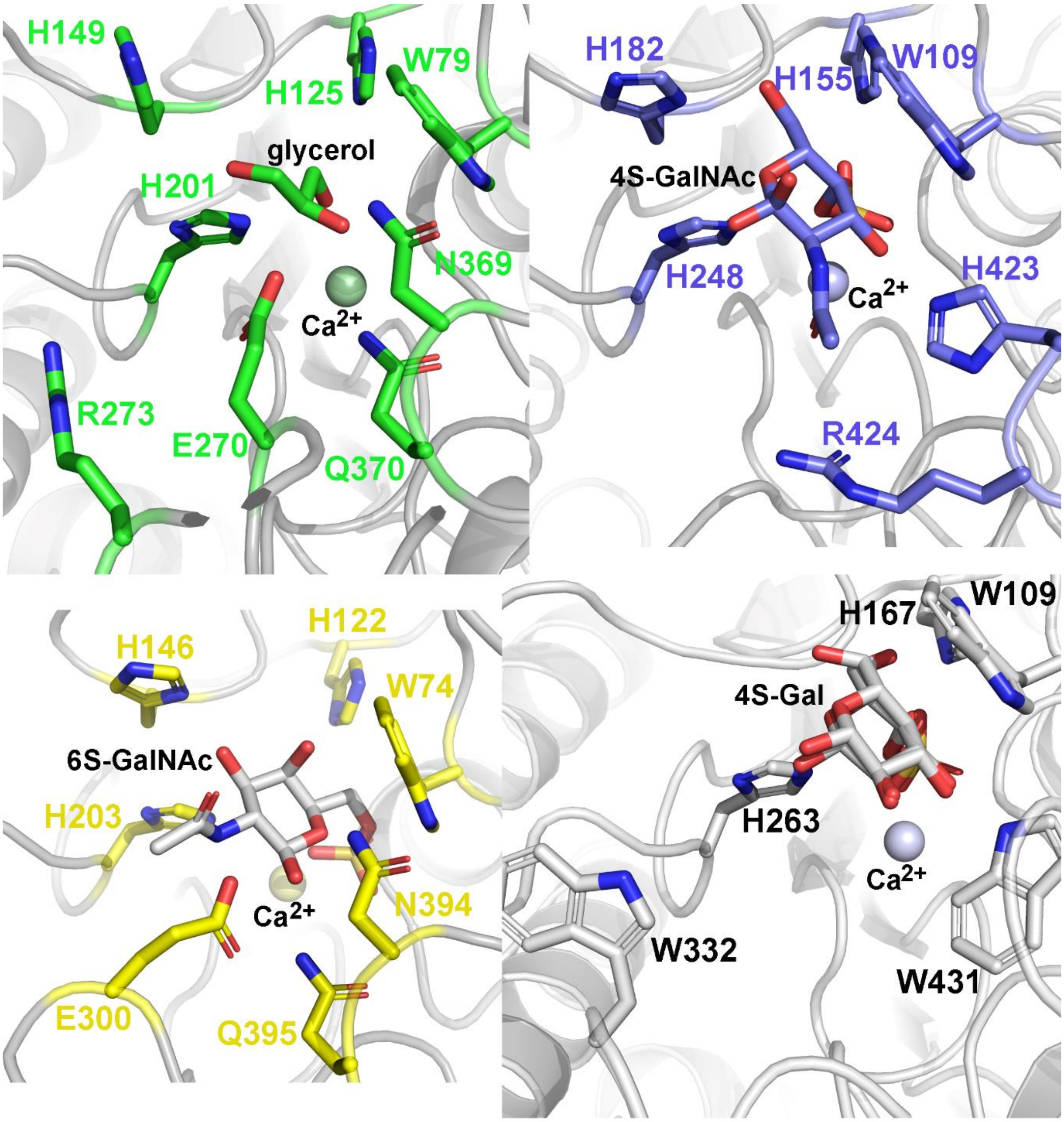

Figure S8. Separated representation of active site residues in superimposed crystal structures of S1_16 sulfatases of Figure 6. Upper left panel (green): active site residues in AMOR_S1_16A; upper right panel (purple): active site residues in BT3057-S1_16 (7OZ9); lower left panel (yellow): active site residues and a docked S6-GalNAc molecule in the sulfatase from *H. hathewayi* (6UST); lower right panel (grey): active site residues in BT3796_S1_16 (7OZA).

Table S1. X-ray crystallography data collection and refinement statistics

Data collection statistics	AMOR_S1_16A
pdb id	9FO1
Beamline	SOLEIL Proxima 2
Wavelength	0.98011
Space group	I23
Resolution	48.6-3.11 (3.33-3.11)
Cell dimension a, b, c (Å)	206.31
α, β, γ (°)	90.0 90.0 90.0
R_{pim}	9.4 (132.8)
Completeness (%)	100.0 (99.5)
<I/σI>	11.4 (1.3)
CC1/2	99.6 (53.0)
Redundancy	42.1 (42.6)
Total reflections	1108546
Unique reflections	26345
Refinement statistics	
R (%)	16.3
R_{free} *(%)*	18.8
RMSD	
Bond lengths (Å)	0.081
Bond angles (°)	1.062
Average *B*-factors (Å^2)	
Protein Chain A	73.7
Water molecules	53.7
Calcium ions	75.7
Glycerol molecule	78.2
Number of atoms	
Protein Chain A	3596
Water molecules	80
Metal ions	2
Glycerol molecule	6
Ramachandran statistics	
Most favored (%)	94.2
Additional allowed (%)	4.9
Disallowed (%)	0.9

Table S2. MALDI-TOF mass spectroscopy analysis of enzymatically hydrolyzed κ-carrageenan (with *Zg*Cgk16A, EC number 3.2.1.83) and TFA-hydrolyzed κ-carrageenan. The abundance of ions matching the theoretical *m/z* of κ-carrageenan hydrolysis products were calculated by normalizing their intensity values relative to: (a) the overall highest intensity value detected between both hydrolysates (*m/z* 811.149, Int: 123526, κ-neocarratetraose, [(G4S+DA)$_2$ + Na]$^-$), and (b) the highest intensity value detected in the TFA hydrolysate (*m/z* 259.048, Int: 22058, galactose-4-sulfate, [G4S]$^-$). *Intensity values of the matrix ions. DP, degree of polymerization; -S, loss of monosodium sulfite ($NaSO_3$). Fully sulfated oligosaccharides are highlighter in bold.

Carrageenase		TFA								
**Found *m/z***	**Relative abundance (%)**	**Found *m/z***	**Relative abundance (%)**		**Assignment**	**Theoretical *m/z***	**DP**	**Assignment 2**	**Calculated *m/z***	**DP**
		**Matrix**								
167.063	*150348	167.074	*155809		[norharmene - H]-	167.0609				
	**κ-carrageenan hydrolysis products**									
	a		a	b						
259.037	6.57	259.048	17.86	100.00	[G4S]$^-$	259.0124	1			
403.068	26.89	403.072	9.87	55.27	[G4S+DA]$^-$	403.0546	2			
547.117	11.22	547.151	5.35	29.96	[DA+G4S+DA]$^-$	547.0969	3	[G4S+DA+G4S - H_2O - S]$^-$	547.0968	3
691.188	7.86	691.224	12.78	71.55	[(G4S+DA)$_2$ - H_2O - S]$^-$	691.1391	4			
811.149	100.00	811.191	6.98	39.12	**[(G4S+DA)$_2$ + Na]$^-$**	811.0885	4			
871.29	7.90	871.326	8.66	48.49	[(G4S+DA)$_2$G4S - 2S]$^-$	871.2025	5			
955.221	1.96	955.267	2.90	16.23	[(DA+G4S)$_2$DA + Na]$^-$	955.1307	5	[(G4S+DA)$_2$G4S - H_2O - S + Na]$^-$	955.1307	7
973.241	3.44	973.288	11.00	61.60	[(G4S+DA)$_2$G4S - S + Na]$^-$	973.1413	5			
1075.282	3.93	1075.249	12.55	70.28	**[(G4S+DA)$_2$G4S + 2Na]$^-$**	1075.0801	5			
997.342	3.04	997.388	8.42	47.13	[(G4S+DA)$_3$ - H_2O - 2S]$^-$	997.2341	6			
1015.342	3.79	1015.394	2.96	16.57	[(G4S+DA)$_3$ - 2S]$^-$	1015.245	6			
1099.29	1.85	1099.343	6.18	34.61	[(G4S+DA)$_3$ - H_2O - S + Na]$^-$	1099.173	6			
1117.304	5.02	1117.357	2.68	14.98	[(G4S+DA)$_3$ - S + Na]$^-$	1117.184	6			

		1201.3	2.57	14.38	$[(G4S+DA)_3 - H_2O + 2Na]^-$	1201.1117	6			
1219.264	5.80	1219.33	1.31	7.32	**$[(G4S+DA)_3 + 2Na]^-$**	1219.1223	6			
1177.449	2.24	1177.496	6.68	37.39	$[(G4S+DA)_3G4S - 3S]^-$	1177.298	7			
1279.413	2.16	1279.468	6.36	35.61	$[(G4S+DA)_3G4S - 2S + Na]^-$	1279.236	7			
1345.376	0.45				$[(DA+G4S)_3+DA -H_2O + 2Na]^-$	1345.154	7			
		1363.427	0.65	3.62	$[(DA+G4S)_3DA + 2Na]^-$	1363.1646	7	$[(G4S+DA)_3G4S -H_2O - S + 2Na]^-$	1363.165	7
1381.384	0.74	1381.442	6.12	34.28	$[(G4S+DA)_3G4S - S + 2Na]^-$	1381.175	7			
1483.633	2.24	1483.422	6.15	34.43	**$[(G4S+DA)_3G4S + 3Na]^-$**	1483.1139	7	$[(G4S+DA)_4G4S - 4S]^-$	1483.393	9
1303.512	1.88	1303.569	5.50	30.79	$[(G4S+DA)_4 - H_2O - 3S]^-$	1303.329	8			
1321.525	6.60				$[(G4S+DA)_4 - 3S]^-$	1321.34	8			
1405.508	0.83	1405.536	3.34	18.68	$[(G4S+DA)_4 - H_2O - 2S + Na]^-$	1405.268	8			
1423.493	3.94	1423.554	1.09	6.11	$[(G4S+DA)_4 - 2S + Na]^-$	1423.279	8			
		1507.522	1.30	7.28	$[(G4S+DA)_4 - H_2O - S + 2Na]^-$	1507.207	8			
1525.461	2.53	1525.547	0.57	3.18	$[(G4S+DA)_4 - S + 2Na]^-$	1525.217	8			
1627.741	10.48	1627.798	1.03	5.79	**$[(G4S+DA)_4 + 3Na]^-$**	1627.1562	8	$[(G4S+DA)_5 - 4S]^-$	1627.435	10
1585.614	1.09	1585.681	4.38	24.53	$[(G4S+DA)_4G4S - 3S + Na]^-$	1585.331	9			
1687.603	0.39	1687.669	3.32	18.60	$[(G4S+DA)_4G4S - 2S + 2Na]^-$	1687.27	9			
1789.871	0.93	1789.94	3.80	21.28	$[(G4S+DA)_4G4S - S + 3Na]$-	1789.209	9	$[(G4S+DA)_5G4S - 5S]^-$	1789.488	11
1891.87	0.33	1891.94	2.64	14.77	**$[(G4S+DA)_4G4S + 4Na]^-$**	1891.1477	9	$[(G4S+DA)_5G4S - 4S + Na]^-$	1891.426	11
1711.72	0.55	1711.769	1.76	9.84	$[(G4S+DA)_5 - H_2O - 3S + Na]^-$	1711.363	10			
1729.724	4.83	1729.797	0.49	2.75	$[(G4S+DA)_5 - 3S + Na]$-	1729.374	10			
1813.734	0.27	1813.807	0.59	3.29	$[(G4+SDA)_5 - H_2O - 2S + 2Na]$-	1813.302	10			
1831.721	2.12				$[(G4S+DA)_5 - 2S + 2Na]$-	1831.312	10			
2036.016	1.11				**$[(G4S+DA)_5 + 4Na]$-**	2035.19	10	$[(G4S+DA)_6 - 4S + Na]$-	2035.469	12
		1993.979	1.46	8.20	$[(G4S+DA)_5G4S - 3S + 2Na]$-	1993.365	11			
2096.223	0.35	2096.259	1.98	11.08	$[(G4S+DA)_5G4S - 2S + 3Na]$-	2095.304	11	$[(G4S+DA)_6G4S - 6S]^-$	2095.583	13
2198.19	0.10	2198.265	1.17	6.55	$[(G4S+DA)_5G4S - S + 4Na]$-	2197.243	11	$[(G4S+DA)_6G4S - 5S + Na]^-$	2197.521	13
		2300.192	0.68	3.82	**$[(G4S+DA)_5G4S + 5Na]$-**	2299.1816	11	$[(G4S+DA)_6G4S - 4S + 2Na]^-$	2299.46	12
		2120.128	0.23	1.31	$[(G4+SDA)_6 - H_2O - 3S + 2Na]$-	2119.397	12			
2138.028	0.47				$[(G4S+DA)_6 - 3S + 2Na]^-$	2137.407	12			
2222.335	0.15	2222.408	0.54	3.03	$[(G4S+DA)_6 - H_2O - 2S + 3Na]$-	2221.336	12	$[(G4S+DA)_7 - H_2O - 6S]^-$	2221.614	14

2240.362	0.64				[(G4S+DA)$_6$ - 2S + 3Na]$^-$	2239.346	12	[(G4S+DA)$_7$ - 6S]-	2239.625	14
		2324.476	0.24	1.34	[(G4S+DA)$_6$ - H_2O - S + 4Na]-	2323.274	12	[(G4S+DA)$_7$ - H_2O - 5S + Na]$^-$	2323.553	14
2342.332	0.29				[(G4S+DA)$_6$ - S + 4Na]$^-$	2341.285	12	[(G4S+DA)$_7$ - 5S + Na]-	2341.564	14
2444.414	0.15				**[(G4S+DA)$_6$ + 5Na]-**	2443.2238	12	[(G4S+DA)$_7$ - 4S + 2Na]$^-$	2443.503	14
2402.574	0.12	2402.665	0.86	4.81	[(G4S+DA)$_6$G4S - 3S + 3Na]-	2401.399	13	[(G4S+DA)$_7$G4S - 7S]$^-$	2401.678	15
		2504.74	0.48	2.70	[(G4S+DA)$_6$G4S - 2S + 4Na]-	2503.338	13	[(G4S+DA)$_7$G4S - 6S + Na]$^-$	2503.617	15
		2606.789	0.28	1.58	[(G4S+DA)$_6$G4S - S + 5Na]-	2605.277	13	[(G4S+DA)$_7$G4S - 5S + 2Na]$^-$	2605.555	15
		2709.137	0.33	1.85	**[(G4S+DA)$_6$G4S + 6Na]-**	2707.2154	13	[(G4S+DA)$_7$G4S - 4S + 3Na]$^-$	2707.494	15
		2528.851	0.17	0.95	[(G4S+DA)$_7$ - H_2O - 3S + 3Na]$^-$	2527.431	14	[(G4S+DA)$_8$- H_2O - 7S]$^-$	2527.709	16
2546.78	0.30				[(G4S+DA)$_7$ - 3S + 3Na]-	2545.441	14	[(G4S+DA)$_8$ - 7S]$^-$	2545.72	16
2648.874	0.16				[(G4S+DA)$_7$ - 2S + 4Na]-	2647.38	14	[(G4S+DA)$_8$ - 6S + Na]$^-$	2647.659	16
2748.997	0.05				[(G4S+DA)$_7$ - S + 5Na]-	2749.319	14	[(G4S+DA)$_8$ - 5S + 2Na]$^-$	2749.598	16
2853.375	0.11				**[(G4S+DA)$_7$ + 6Na]-**	2851.2577	14	[(G4S+DA)$_8$ - 4S - 3Na]$^-$	2851.536	16
		2811.202	0.13	0.74	[(G4S+DA)$_7$G4S - 3S + 4Na]-	2809.433	15	[(G4S+DA)$_8$G4S - 7S + Na]$^-$	2809.712	17
		2913.376	0.10	0.56	[(G4S+DA)$_7$G4S - 2S + 5Na]-	2911.372	15	[(G4S+DA)$_8$G4S - 6S + 2Na]$^-$	2911.65	17
		3016.346	0.11	0.64	[(G4S+DA)$_7$G4S - S + 6Na]-	3013.311	15	[(G4S+DA)$_8$G4S - 5S + 3Na]$^-$	3013.589	17
		3117.613	0.10	0.54	**[(G4S+DA)$_7$G4S + 7Na]$^-$**	3115.2493	15	[(G4S+DA)$_8$G4S - 4S + 4Na]$^-$	3115.528	17
2953.311	0.04				[(G4S+DA)$_8$ - 3S + 4Na]$^-$	2953.475	16	[(G4S+DA)$_9$- 7S + Na]$^-$	2953.754	18
3058.498	0.05	259.048			[(G4S+DA)$_8$ - 2S + 5Na]$^-$	3055.414	16	[(G4S+DA)$_9$- 6S + 2Na]$^-$	3055.693	18

Table S3. ^{1}H and ^{13}C chemical shifts for TFA-hydrolyzed κ-carrageenan treated with AMOR_S1_16A (10 µM AMOR_S1_16A and 2 mg/mL substrate under optimal reaction conditions) acquired in 99.9% D_2O at 25 °C on 800 MHz spectrometer.

		Carbon and proton chemical shifts (ppm)						
**Residue**		**1**	**2**	**3**	**4**	**5**	**6a**	**6b**
**G4S**	3)-β-D-Gal*p*-(4SO3^{-})-(1							
		104.7	71.6	80.4	75.9	77	63.5	
		4.63	3.57	3.98	4.83	3.79	3.78	
**G4SREa**	3)-α–D-Gal*p*-(4SO3^{-})							
		94.6	69.5	77.4	77.2	72.4	63.8	63.8
		5.29	3.89	4.15	4.87	4.16	3.71	3.77
**G4SREb**	3)-β-D-Gal*p*-(4SO3^{-})							
		98.8	72.8	80.5	76.1	76.8	-	
		4.62	3.57	3.95	4.81	3.75	-	
**G4SNRE**	β-D-Gal*p*-(4SO3^{-})-(1							
		104.9	73.3	74.2	79.1	77.2	63.5	
		4.59	3.49	3.78	4.65	3.84	3.78	
**GNRE**	β-D-Gal*p*-(1							
		105.1	73.3	75.2	71.2	77.9	63.6	63.6
		4.54	3.47	3.63	3.9	3.7	3.77	3.71
**DA**	4)-3,6-anhydro-α-D-Gal*p*-(1							
		96.8	71.8	81.4	80.6	78.9	71.7	71.7
		5.08	4.11	4.5	4.57	4.62	4.2	4.03
**DA'**	4)-3,6-anhydro-α-D-Gal*p*-(1							
		-	-	81.5	80.4	-	-	
		-	-	4.48	4.57	-	-	

Table S4. Results of the structural superimposition of AMOR_S1_16 to the coordinates of S1 sulfatase structures 6UST, 7OZA and 7OZ9, top best hits, identified using Dali. The root-mean square deviation (rmsd) was calculated for the Cα atoms using SSM-superimposition with COOT, and the number of structurally aligned residues with respect to the total number are given.

	RMSD (Å)	Number of residues	Number of residues aligned
6UST/AMOR_S1_16	0.962	463/451	417
6UST/7OZA	1.400	463/487	407
6UST/7OZ9	1.440	463/482	394
7OZA/AMOR_S1_16	1.325	487/451	405
7OZA/7OZ9	1.258	487/482	427
7OZ9/AMOR_S1_16	1.403	482/451	389
